# Promoting couples’ resilience to relationship obsessive compulsive disorder (ROCD) symptoms using a CBT-based mobile application: A randomized controlled trial

**DOI:** 10.1016/j.heliyon.2023.e21673

**Published:** 2023-10-28

**Authors:** Milana Gorelik, Ohad Szepsenwol, Guy Doron

**Affiliations:** a Baruch Ivcher School of Psychology, Reichman University (IDC), Herzliya, Israel; b The Max Stern Yezreel Valley College, Emek Yezreel, Israel

**Keywords:** Obsessive-compulsive disorder (OCD), Relationship obsessive-compulsive disorder (ROCD), Cognitive-behavioral therapy, Mobile app, Internet-based interventions

## Abstract

Relationship Obsessive Compulsive Disorder (ROCD) is a disabling form of obsessive-compulsive disorder (OCD) centering on interpersonal relationships. Previous findings suggest ROCD symptoms are particularly detrimental to romantic relationships. In this randomized controlled trial (RCT), we assessed influence a CBT-based mobile application used by both partners on resilience to ROCD symptoms, cognitions, and relationship dissatisfaction. The app consists of brief, daily exercises targeting OCD symptoms, related cognitions and attachment insecurities. Heterosexual couples (*N*_couples_ = 103; *M*age = 26.15) were randomly assigned to individually use a mobile application for 15 days (*n* = 49 couples) or to a control group (*n* = 54 couples). All participants completed questionnaires at baseline (T1), 15 days from baseline (T2), and 45 days from baseline (T3). All couples also underwent an ROCD resilience task at T2. Intention-to-treat analyses revealed that, in contrast to the control group, couples who used the app exhibited enhanced resilience in the resilience task, as well as measures of ROCD symptoms, cognitions, and relationship dissatisfaction. These observed effects persisted even at the 1-month follow-up. Concurrent use of brief mobile delivered cognitive training by both romantic partners may foster resilience in romantic couples.

## 1 Introduction

Healthy romantic relationships have numerous benefits [1], including better mental [1] and physical health [2], increased subjective wellbeing [3] and higher self-esteem [4]. Romantic relationships also protect mental health and well-being during disruptive and stressful life events [5,6]. However, pre-existing vulnerabilities, such as increased obsessive-compulsive disorder (OCD) symptoms, could make it difficult to maintain healthy relationships [7]. One presentation of OCD that is especially relevant to couple functioning is *Relationship Obsessive-Compulsive Disorder* (ROCD [[8], [9], [10]]).

ROCD has two main symptom presentations that tend to co-occur [11]: relationship-centered and partner-focused obsessive-compulsive disorder (OCD) symptoms [8]. Relationship-centered OCD symptoms include doubts and preoccupation that focus on the “rightness” of the relationship, the intensity of one's feelings towards the partner, and the nature of the partner's feeling toward oneself [8,12]. Partner-focused ROCD symptoms involve debilitating doubts and preoccupations centered on the perceived flaws and shortcomings of one's relationship partner spanning various domains, such as appearance, social functioning, morality, intelligence, general competence, and trustworthiness [13,14].

ROCD symptoms have been reported across the world including in the US, and the UK (e.g. Ref. [15]), Spain (e.g. Ref. [16]), Italy (e.g. Refs. [17,18]), Turkey (e.g. Ref. [19]), Iran (e.g. Ref. [20]) and Israel (e.g. Refs. [12,14]). Most previous studies have focused on ROCD within romantic relationships. However, ROCD symptoms can manifest within different types of relationships (e.g., romantic, parent-child, God-individual) with significant consequences [8,21,22].

Individuals with ROCD symptoms may describe being preoccupied with thoughts (e.g., “Is my partner funny enough?“), images (e.g., Image of the partner looking unattractive) or urges (i.e., to leave their partner) relating to the suitability of the partner or the relationship [23]. As in other presentations of OCD, these intrusive experiences are unwanted and often unexpected [24].

In romantic relationships, empirical evidence suggests that ROCD symptoms have detrimental effects on personal and dyadic well-being in clinical and non-clinical populations [e.g., 12, 14, 25]. Notably, the presence of ROCD symptoms has been correlated with increased anxiety, negative affect, OCD symptoms, and lower self-esteem [12,14]. ROCD symptoms have also been linked with relationship-related difficulties, including poor relationship and sexual satisfaction, low commitment levels, insecure attachment, and excessive jealousy [8,9,[12], [13], [14],^24^].

According to cognitive behavioral therapy (CBT) models of OCD, unwanted intrusive experiences such as thoughts, images, or urges are common [e.g., [26]]. Individuals with OCD, however, tend to attribute catastrophic interpretations to these intrusions [[27], [28], [29], [30]]. It has been proposed that maladaptive beliefs (e,g., inflated responsivity and importance of thoughts) contribute to the increased likelihood of such catastrophic appraisals [27,28].

Similarly, one CBT model of ROCD proposed by Doron and colleagues [8] stipulates that maladaptive beliefs associated with OCD and relationship-specific maladaptive beliefs (e.g., catastrophizing the consequences of being in an incompatible relationship [17,25]) lead to catastrophic appraisals (e.g., “This relationship is the biggest mistake I've made”) of common relationship-related intrusions (e.g., “I'm bored with my partner”). Attachment insecurities and associated dysfunctional emotional regulation strategies are then activated leading to further escalation of distress and the use of counterproductive behaviors resulting in obsessive compulsive symptoms [31]. This model also proposes pre-existing personality traits (e.g., narcissistic traits [18,32]) and self-vulnerabilities [33] make individuals more susceptible ROCD symptoms.

CBT, alongside exposure and ritual prevention, is recognized as the primary treatment approach for OCD [34,35]. CBT-based interventions help reduce OCD symptoms by challenging and reducing maladaptive beliefs and associated behaviors [24,36,37]. Despite its efficacy, however, CBT is vastly underused among individuals with OCD for a variety of reasons, including treatment expenses, stigma, and challenges in accessing trained therapists [38,39]. One potential means to overcome many of these barriers comes in the form of internet-delivered cognitive behavioral therapy (iCBT) and mobile-delivered CBT applications (CBT-apps). These forms of intervention have been increasingly researched in recent years and have proven to increase accessibility to CBT treatments [40,41].

GGtude is a CBT‐based mobile platform with significant research support for. This platform includes modules targeting a wide variety of psychological symptoms including anxiety, depression, OCD, ROCD, self-esteem and body image-related symptoms [16,[42], [43], [44], [45], [46], [47]]. Across nine randomized controlled trials (RCTs) conducted in various countries (US, Spain, Italy, Turkey & Israel) daily use of apps from the GGtude platform for a duration of two to four weeks (averaging 3 min per day) has consistently demonstrated significant beneficial effects in non-clinical [43,47], subclinical [45,[48], [49], [50], [51]] and clinical samples [44].

The GGtude platform uses several mechanisms to increase the relative activation of adaptive cognitions, making them more readily accessible compared to maladaptive ones [42,52]. For instance, daily categorizing of cognitions using contrasting bodily movements (i.e., swiping upwards to discard maladaptive cognitions and swiping downwards to embrace adaptive ones; see detailed description below) may increase users' awareness of their cognitions and lead to clearer signals regarding their relevance to users' own mental health goal (i.e. embodied cognition [53]). Such exercises also include daily exposure to adaptive cognitions, thereby bolstering users' capacity to generate and retrieve adaptive cognitions. Succinct psychoeducation scripts (e.g., “Feeling doubt is normal. Pull statements that encourage tolerating doubt rather than fearing it”) may further motivate and facilitate users’ comprehension of fundamental CBT principles [54].

In the current study, the ROCD module of “OCD.app - Anxiety, mood & sleep” of the GGtude platform (GGRO) was used concurrently by both romantic partners. Previous RCTs have shown GGRO to be effective in reducing ROCD symptoms and related cognitions. For example, an RCT with 50 individuals showing subclinical levels of ROCD showed that app use is associated with lower ROCD symptoms and OCD cognitions, and higher self-esteem compared to the control group. Moreover, the Reliable Change Index (RCI [55]), indicated a reliable change in ROCD symptoms for a substantial portion (42–52 %) of participants [45].

The primary objective of the present study was to assess the efficacy of concurrent use of GGRO by both romantic partners in protecting them against increased ROCD symptoms and cognitions, depression symptoms, attachment insecurity, relationship dissatisfaction, and sexual dissatisfaction. We conducted an RCT comparing romantic couples who used the relationship module of ‘GG OCD, Anxiety, mood and sleep’ app (GGRO) for 15 days to a control group of romantic couples who did not use it, across three time points (pre-intervention, post-intervention, and one-month follow up [45 days from baseline]).

We hypothesized that compared to the control group, couples who used the GGRO module would show fewer ROCD symptoms, greater relationship satisfaction, and better sexual functioning immediately following app use and at the one-month follow-up. We also expected that couples using the app will show decreased ROCD vulnerabilities and maintaining factors such as obsessive-compulsive related beliefs, relationship catastrophizing beliefs, and attachment insecurities. Further, we hypothesized that couples who used GGRO would show fewer ROCD symptoms following an ROCD resilience task (see description in the method section), compared to the couples who did not use the app.

To the best of our knowledge, this is the first study to examine the effects of concurrent use of an mHealth mental health app by both romantic partners. This is also the first study to use a resilience task and change in baseline measures as indicators of the potential benefits of using a mental health app in fostering resilience to OCD-related symptoms and cognitions.

## 2 Method

### 2.1 Participants

**Fig. 1:**
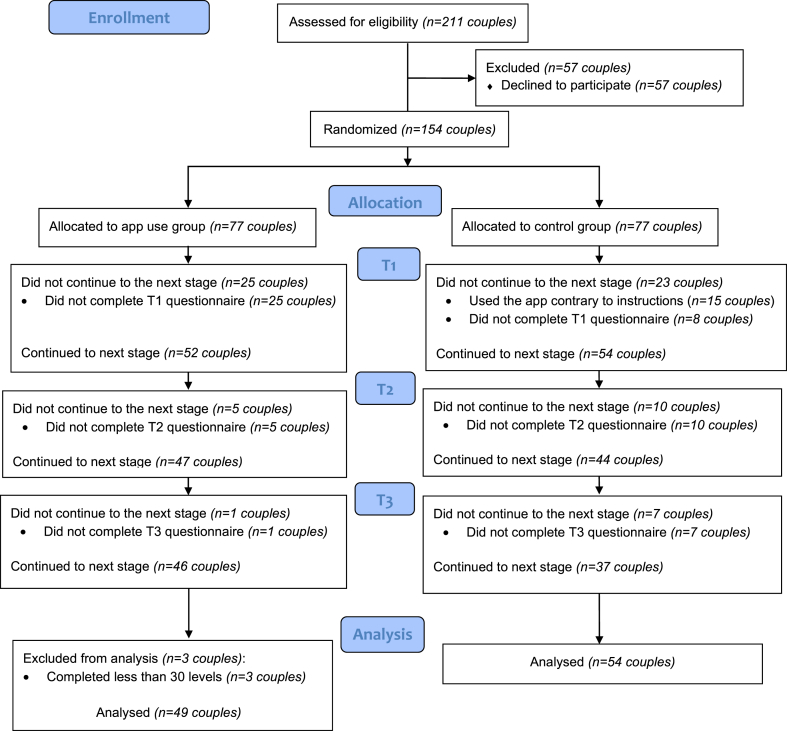
Flow diagram of participants through the study.

One hundred fifty-four couples who met the inclusion criteria (ages 18–65, in a committed monogamous relationship for at least 4 months, a mobile device compatible with GGRO) enrolled for the study. The couples were recruited via publications on online social platforms, such as Facebook and Instagram, in which they were invited to participate in a study that examines romantic relationships. As compensation, participants were entered into a drawfor a prize (a spa day including breakfast for two valued at 500 ILS). Thirty-three couples chose to withdraw before the initial evaluation and 15 couples failed to adhere to the study's guidelines (e.g., used the app in the control condition; see Fig. 1).

Of the 103 couples analyzed, 24.3 % (n = 25) females and 17.5 % (n = 18) male participants met the ROCD symptoms threshold criteria on either the PROCSI (PROCSI>0.71 [56]) or the ROCI (ROCI>1.79 [56]). Also, of the couples analyzed, 23 couples dropped out during the study (6 couples from the experimental group and 17 couples from the control group; see Fig. 1). The study obtained ethical approval from the Reichman University ethics committee (Ethical clearance number: P_2,019,240).

Power analysis conducted with G*Power [57] indicated that the sample size provides 95 % power to detect medium-small (*f* = 0.16) between-within (e.g., group × time) interactions at the univariate level.

### 2.2 Measures

*The Relationship Obsessive–Compulsive Inventory* (ROCI [12]) is a 12-item self-report measure tapping relationship-centered ROCD symptoms. Each item is rated on a 5-point scale (0 = *not at all*, 4 = *very much*). The measure demonstrated excellent internal consistency (Cronbach's *α* = 0.93 [12]). In the current study, at all different measurement times, the questionnaire demonstrated reliability ranging from very good (Cronbach's *α* = 0.84; at T3) to excellent (Cronbach's *α* = 0.91; at T2).

*The Partner-Related Obsessive–Compulsive Symptoms Inventory* (PROCSI [14]) is a 24-item self-report measure of partner-focused ROCD symptoms in six character domains: appearance, morality, sociability, intelligence, emotional stability, and general competence. Each item is rated on a 5-point scale (0 = *not at all*, 4 = *very much*). The measure has excellent internal consistency (Cronbach's *α* = 0.95 [14]). In the current study, the internal consistency of the scale was excellent across measurement times (Cronbach's *α* ranging from 0.92 at T2 and T3 to 0.90 at T1).

*The Relationship Catastrophization Scale* (ReCats [25]) is an 18-item self-report measure designed to tap into three relational belief domains represented by six items each, including overestimation of the negative consequences of (1) being alone (2) separating with one's partner, and (3) being in the wrong relationship. Each item is rated on a 7-point scale (1 = *disagree very much*, 7 = *agree very much*). The measure demonstrated adequate internal consistency (Cronbach's *α*s ranging from 0.79 to 0.87 [25]). In the current study, the internal consistency of the scale was very good across measurement times (Cronbach's *α* ranging from 0.89 at T1 and T2 to 0.85 at T3).

*The Relationship Assessment Scale* (RAS [58]) in its short version [59], is a four-item measure that assesses the perceived level of relationship satisfaction (“e.g., how much do you love your romantic partner?”), using a 7-point scale ranging from 1 (*not at all*) to 5 (*extremely*). The measure has demonstrated very good internal consistency (Cronbach's *α* = 0.86 [59]). In the current study, the questionnaire demonstrated reliability ranging from very good (Cronbach's *α* = 0.82; at T1) to moderate (Cronbach's *α* = 0.64; at T3).

*The Changes in Sexual Functioning Questionnaire-14* (CSFQ-14 [60]) is an abbreviated version of the 36-item Changes in Sexual Functioning Questionnaire (CSFQ [61]). This instrument provides a global measure for sexual enjoyment/pleasure, desire, arousal, and orgasm. Each item is rated on a 5-point Likert scale. The measure demonstrated very good internal consistency (Cronbach's *α* of 0.90 for the female version and 0.89 for the male version [60]). In this study, the internal consistency of both the male and female versions of the questionnaire ranged from good (Cronbach's *α* = 0.72 for the male version, and *α* = 0.76 for the female version; at T1) to moderate (Cronbach's *α* = 0.66 for both versions; at T3).

*The Experiences in Close Relationships Scale* (ECR [62]), in its short version (ECR-S [63]), includes 12 items: six assessing attachment anxiety and six assessing attachment avoidance. Participants were asked to rate each item on a 7-point scale (1 = *disagree strongly*, 7 = *strongly agree*). The ECR-S demonstrated good internal consistency for the anxiety scale (Cronbach's *α* = .78) and very good internal consistency for the avoidance scale (Cronbach's *α* = .84 [63]). In this study, the internal consistency of the anxiety and avoidance scales ranged from very good (Cronbach's *α* = .87, *α* = .82 respectively; at T1) to good (Cronbach's *α* = .74, *α* = .73 respectively; at T3).

*The Obsessional Beliefs Questionnaire-20 items* (OBQ-20 [64]) is an abbreviated version of the 44-item Obsessive Beliefs Questionnaire-Revised [28], a self-report measure of pan-situational cognitions associated with OCD. The 20-item OBQ assesses four dysfunctional belief domains commonly associated with OCD: overestimation of threat, inflated responsibility for harm, over-importance/control of thoughts, and perfectionism/intolerance of uncertainty. Each item is rated on a 7-point scale (1 = *disagree very much*, 7 = *agree very much*). Cronbach's *α*s across subscales ranging from 0.81 to 0.93 have been found in large non-clinical samples [64]. In the current study, the internal consistency of the scale was very good across measurement times (Cronbach's *α*s ranging from 0.89 at T1 and T2 to 0.86 at T1).

*The short version of the Depression, Anxiety, Stress Scale*s (DASS-21 [65]) is a 21-item self-report questionnaire listing negative emotional symptoms. The scale is divided into three subscales measuring depression, anxiety, and stress. In this study, only the depression subscale (7 items) was used. The Depression subscale assesses dysphoria, hopelessness, devaluation of life, self-deprecation, lack of interest/involvement, anhedonia, and inertia on a 4-point scale (0 = *the statement is not true about me at all*, 3 = *the statement is true to a great extent or most of the time*). The depression subscale demonstrated excellent internal consistency (Cronbach's *α* = 0.91 [65]). In the current study, the internal consistency of the scale was very good across measurement times (Cronbach's *α*s ranging from 0.86 at T1 to 0.83 at T3).

*The Relationship Obsessive–Compulsive Inventory* scenarios (ROCIscen [31]) measure ROCD symptoms that are centered on aspects of the romantic relationship. The inventory includes nine scenarios, such as: “You are about to meet your partner for lunch, suddenly a thought raises that you do not truly love him\her. To what extent do you feel an urge to do something about that?”. Each item is rated on a 9-point scale (0 = *not at all*, 9 = *very much*). All scenarios were averaged to create a single score. The measure demonstrated excellent internal consistency (Cronbach's *α* = 0.97 [31]). In the current study, the questionnaire was used at T2 (Cronbach's *α* = 0.84).

### 2.3 Intervention

**Fig. 2:**
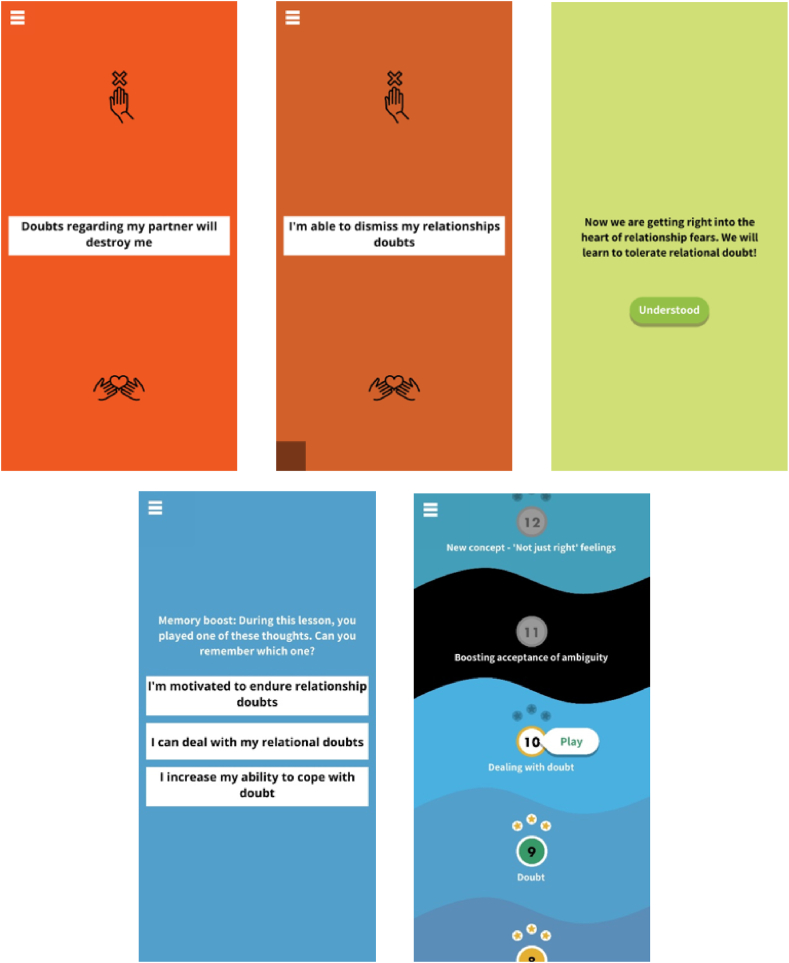
GGRO: Relationship Doubts screenshots.

The relationships module of the GGtude platform (GGRO) was designed with the aim of challenging dysfunctional beliefs that underlie ROCD symptoms and related phenomena [16]. First-time users are guided through a brief tutorial explaining the influence of one's self-talk on mood. Subsequently, they learn how to use short, game-like interactions to make their self-talk more helpful. Each training session comprises self-statements that are either consistent (e.g., “An imperfect relationship is worthless”) or inconsistent (e.g., “Imperfect is interesting”) with a specific maladaptive belief (e.g. perfectionism). They are instructed to discard maladaptive self-statements by “throwing” them away from themselves (i.e., upward motion) and to “embrace” helpful self-statements by pulling them towards themselves (i.e., downward motion; see Fig. 2).

Upon the introduction of a new set of levels addressing a particular maladaptive belief, a screen explaining the rationale for challenging this specific belief is presented. For instance, before learning to challenge relationship doubts, the following statement is presented: “Now we are getting right into the heart of the relationship fears. We will learn to tolerate relational doubt!”. Following the completion of a level, a brief memory quiz ensues, requiring participants to identify one out of three presented OCD-challenging statements that was featured in the recently completed level. After completing three levels, a screen displays the following message: “You've reached the recommended amount of training for today. To get the best results, continue practicing tomorrow”, prompting participants to conclude their app session and resume their training the following day. Notably, previous studies have demonstrated the app's effectiveness in reducing ROCD symptoms [16,45,47].

### 2.4 Procedure

Prior to the beginning of the study, all participants received detailed information regarding the terms of participation and provided online informed consent under Reichman University standards. The entire study was conducted online using Qualtrics. Responses were collected and stored anonymously and then downloaded for analysis.

**Fig. 3:**
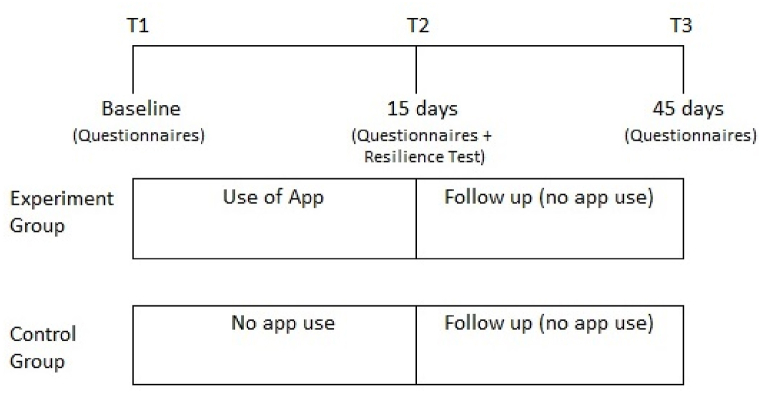
Study design with both groups.

Couples were assigned randomly to either the experimental group or the control group. They downloaded the application and completed the pre-treatment evaluation (T1) on Qualtrics. Reminders to complete T2 and T3 evaluations were sent via email. The study spanned a total duration of 45 days, during which measurements were taken at three different times (See Fig. 3).

Prior to T1, each couple received a random ID number and completed the consent form and a short demographic questionnaire. They were then asked to complete the Hebrew validated versions of the measures mentioned above, to assess the levels of ROCD symptoms, mood, attachment, OC and relationship beliefs and cognitions, sexual functioning, and relationship satisfaction. Then, couples in the control group were asked not to use the application until the end of the study, whereas couples in the experimental group were instructed to use the app daily for a period of 15 days and complete three training levels a day (approximately 3 min), preferably at the same time during each day. Couples in the experimental group were instructed to allow push notifications and were contacted by email once a week to improve compliance with the task.

Fifteen days after the baseline evaluation (T2), all couples were asked to answer the same battery of questionnaires, followed by a resilience task. During the resilience task, a bogus short article with relationship-centered triggering content was presented, after which participants completed a short memory quiz to ensure that they have read the article and paid attention to the content. This was followed by an evaluation of their current relationship-centered ROCD symptoms (ROCD scenarios, see measures section). All participants were then informed that the article was fake, in order to prevent any long-term distress that could have been caused by the content of the article. The same battery of questionnaires as in T1 and T2 was employed after a month for the final follow-up assessment (T3). Finally, all participants were thanked for their participation and given a short debriefing about the goal of the study and information about ROCD symptoms.

### 2.5 Statistical analysis

Statistical analyses were carried out using IBM SPSS. Descriptive statistics were computed to report means, standard deviations, and frequencies. A series of 2 (Group: experimental, control) × 2 (Gender: male, female) between-within analysis of variance (ANOVA) tests and *χ*^*2*^ tests were performed to assess baseline differences between study groups (experimental vs. control). Gender was included as a within-couple variable in the ANOVAs to control for gender effects and group × gender interactions. For variables that were measured at the individual level, separate *χ*^*2*^ tests were performed for men and women. Another set of 2 (Completion status: completed, dropped out) × 2 (Gender: male, female) between-within analysis of variance (ANOVA) tests were performed to assess baseline differences between couples who completed the study and those who dropped out.

To avoid over-optimistic estimates of the efficacy of the training [66], an intention-to-treat analysis was conducted using Multivariate Imputation with Chained Equations (MICE [67]). Multiple imputation methods follow 3 steps: a) Imputing – repeated over several iterations (i) as opposed to a single imputation. b) Analyzing – each iteration of the dataset is analyzed, leading to a distribution of i statistics, 1 per dataset. c) Pooling – the i results are pooled into one estimate [68].

Training effects between groups were tested using an omnibus 2 (Group: experimental, control) × 3 (Time: T1, T2, T3) × 2 (Gender: male, female) between-within-within multivariate analysis of variance (MANOVA) on all tested variables. When significant multivariate effects emerged, univariate ANOVAs and Bonferroni post-hoc comparisons were performed to examine their source. Additionally, a 2 (Group: experimental, control) × 2 (Gender: male, female) between-within ANOVA was employed to assess differences between the experimental and control groups in current relationship-centered ROCD symptoms following the resilience test at T2.

## 3 Results

### 3.1 Baseline differences in demographic characteristics between study groups

A total of 103 couples were included in the analyses. To examine whether study groups differed in their demographic characteristics at baseline (T1), three 2 (Group: experimental, control) × 2 (Gender: male, female) between-within analyses of variance (ANOVA) and six chi-square tests were conducted. The results are shown in Table 1.

**Table 1:** Descriptive statistics and comparisons between experimental group and control group in demographic variables.

		Experimental (n = 49 couples) *M(SD)*	Control (n = 54 couples) *M(SD)*	*F* (group)/*χ*^*2*^	*Df*	*p*	*η*_*p*_^*2*^/Phi
Age		26.68 (5.67)	25.66 (3.43)	1.41	1	.24	.01
Relationship duration (months)		45.44 (36.71)	45.55 (36.63)	.00	1	.99	.000
Family status^1,2^	Married Unmarried	28.6 % 71.4 %	16.7 % 83.3 %	2.10	1	.15	.14
Cohabitation^3,2^	No Yes	42.9 % 57.1 %	42.6 % 57.4 %	.001	1	.98	.003
Children^3^ – men	No Yes	81.6 % 18.4 %	90.7 % 9.3 %	1.82	1	.18	.13
Children^3^ – women	No Yes	81.6 % 18.4 %	92.6 % 7.4 %	2.80	1	.09	.17
Religiosity^4^ - men	Secular Non-secular	75.5 % 24.5 %	72.2 % 27.8 %	.14	1	.71	.04
Religiosity^4^ – women	Secular Non-secular	63.3 % 36.7 %	75.9 % 24.1 %	1.96	1	.16	.14
Years of education		14.12 (2.10)	13.44 (1.94)	4.68	1	.03	.04

Note: ^1^ nominal variable transformed into dichotomous variable.

^2^one score was computed for each couple.

^3^variables inserted in a dichotomous manner.

^4^ordinal variable transformed into a dichotomous variable.

As seen in Table 1, couples in the experimental group did not differ from couples in the control group in terms of age, relationship duration, family status, cohabitation status, children and level of religiosity. However, couples in the experimental group were more educated than couples in the control group.

### 3.2 Baseline differences between study groups in tested variables

To examine whether both study groups differed regarding the tested variables at baseline measurement (T1), nine 2 (Group: experimental, control) × 2 (Gender: male, female) between-within analyses of variance (ANOVA) were conducted. The results are presented in Table 2.

**Table 2:** Descriptive statistics and comparisons between experimental group and control group in symptom, relationship, and sexual experience scales.

	Experimental (n = 49 couples) *M(SD)*	Control (n = 54 couples) *M(SD)*	*F* (group)	*Df*	*p*	*η*_*p*_^*2*^
ROCI	.49 (.53)	.52 (.51)	.07	1	.79	.001
PROCSI	.43 (.44)	.44 (.40)	.02	1	.88	.000
ReCats	3.59 (1.14)	3.89 (1.09)	3.07	1	.08	.03
OBQ-20	2.95 (.93)	3.08 (.88)	1.12	1	.29	.01
DASS-7	.38 (.44)	.44 (.53)	.55	1	.46	.005
ECR-S - anxiety	3.42 (1.48)	3.24 (1.43)	.78	1	.38	.008
ECR-S - avoidance	3.66 (1.13)	3.72 (1.25)	0.17	1	.68	.002
RAS	6.29 (.73)	6.27 (.63)	.02	1	.88	.000
CSFQ-14	48.67 (6.38)	47.71 (6.62)	1.20	1	.28	.01

Note: *DASS-7: Depression anxiety stress scale; ECR-S: short form of Experience in close relationship scale; OBQ-20: short form of Obsessional Beliefs Questionnaire; RAS: Relationship assessment scale; ReCats: Relationship Catastrophization Scale; ROCI: Relationship Obsessive–Compulsive Inventory; PROCSI: Partner-Related Obsessive–Compulsive Symptoms Inventory; CSFQ-14: short form of Changes In Sexual Function Questionnaire*.

As seen in Table 2, couples from both study groups did not differ in their ROCD symptoms (partner-focused and relationship-centered), OC and relationship beliefs and cognitions, level of depression, attachment orientation, relationship satisfaction, and sexual functioning.

3.3 Baseline differences in demographic characteristics between couples who completed all assessments and couples who dropped out.

In order to examine whether couples who completed all assessments differed from couples who dropped out during the study, regarding their demographic characteristics at baseline (T1), three 2 (Completion status: completed, dropped out) × 2 (Gender: male, female) between-within analyses of variance (ANOVA) and six chi-square tests were conducted. The results are shown in Table 3.

**Table 3:** Descriptive statistics and comparisons between couples who completed all assessments and couples who dropped out in demographic variables.

		Completed (n = 80 couples) *M(SD)*	Dropped out (n = 23 couples) *M(SD)*	*F* (group)/*χ*^*2*^	*Df*	*p*	*η*_*p*_^*2*^/Phi
Age		26.34 (4.99)	25.48 (3.24)	.68	1	.41	.007
Relationship duration (months)		45.49 (36.84)	45.52 (36.06)	.00	1	1.00	.000
Family status^1,2^	Married Unmarried	23.8 % 76.3 %	17.4 % 82.6 %	.42	1	.52	.06
Cohabitation^3,2^	No Yes	42.5 % 57.5 %	43.5 % 56.5 %	.01	1	.93	.008
Children^3^ - men	No Yes	83.8 % 16.2 %	95.7 % 4.3 %	2.16	1	.14	.15
Children^3^ - women	No Yes	85.0 % 15.0 %	95.7 % 4.3 %	1.84	1	.18	.13
Religiosity^4^ - men	Secular Non-secular	72.5 % 27.5 %	78.3 % 21.7 %	.31	1	.58	.06
Religiosity^4^ - women	Secular Non-secular	75.0 % 25.0 %	52.2 % 47.8 %	4.42	1	.04	.21
Years of education		13.94 (2.01)	13.13 (2.03)	4.55	1	.04	.04

Note: ^1^ nominal variable transformed into dichotomous variable.

^2^one score was computed for each couple.

^3^variables inserted in a dichotomous manner.

^4^ordinal variable transformed into a dichotomous variable.

As seen in Table 3, couples who completed all assessments did not differ from couples who dropped out regarding most variables. However, women who completed all assessments were more secular than women who dropped out. In addition, couples who completed all assessments were more educated than couples who dropped out.

### 3.3 Differences between couples who completed all assessments and couples who dropped out regarding tested variables

To examine whether couples who completed all assessments differed from couples who dropped out during the study, regarding the tested variables at baseline (T1), nine 2 (Group: completed, dropped out) × 2 (Gender: male, female) between-within analyses of variance (ANOVA) were conducted. The results are presented in Table 4.

**Table 4:** Descriptive statistics and comparisons between couples who completed all assessments and couples who dropped out in tested variables at baseline.

	Completed (n = 80 couples) *M(SD)*	Dropped out (n = 23 couples) *M(SD)*	*F* (group)	*Df*	*p*	*η*_*p*_^*2*^
ROCI	.49 (.50)	.56 (.57)	.44	1	.51	.004
PROCSI	.44 (.45)	.40 (.33)	.37	1	.54	.004
ReCats	3.67 (1.12)	4.01 (1.06)	2.84	1	.10	.03
OBQ-20	2.95 (.88)	3.23 (.96)	3.84	1	.05	.04
DASS-7	.42 (.51)	.38 (.39)	.23	1	.63	.002
ECR-S- anxiety	3.25 (1.46)	3.57 (1.39)	1.78	1	.19	.02
ECR-S- avoidance	3.80 (1.20)	3.33 (1.11)	6.36	1	.01	.06
RAS	6.26 (.69)	6.34 (.64)	.31	1	.58	.003
CSFQ-14	48.05 (6.76)	48.59 (5.60)	.26	1	.61	.003

Note: *DASS-7: Depression anxiety stress scale; ECR-S: short form of Experience in close relationship scale; OBQ-20: short form of Obsessional Beliefs Questionnaire; RAS: Relationship assessment scale; ReCats: Relationship Catastrophization Scale; ROCI: Relationship Obsessive–Compulsive Inventory; PROCSI: Partner-Related Obsessive–Compulsive Symptoms Inventory; CSFQ-14: short form of Changes In Sexual Function Questionnaire*.

As seen in Table 4, couples who completed all assessments and couples who dropped out were similar regarding most studied variables However, couples who completed all assessments were higher in attachment avoidance compared with couples who dropped out.

### 3.4 Main analyses

To assess whether couples using GGRO showed improved functioning over time on the measured variables relative to couples not using the app, we conducted an omnibus 2 (Group: experimental, control) × 3 (Time: T1, T2, T3) × 2 (Gender: male, female) between-within-within multivariate analysis of variance (MANOVA) on all assessed variables. To correct for univariate multiple comparisons, we used a false discovery rate (FDR [69,70]) correction (p < .05). The FDR adjusts the criterion alpha level for significance based on the number of statistical tests conducted that fail to reach an increasingly stringent probability level. In this study, there were two tests that were not significant, and therefore the criterion alpha was adjusted to 0.044.

The MANOVA revealed significant multivariate group (Pillai's Trace = 0.23, *F*(9, 93) = 3.08, *p* = .003, *η*_*p*_^*2*^ = 0.23), time (Pillai's Trace = 0.57, *F*(18, 84) = 6.14, *p* < .001, *η*_*p*_^*2*^ = 0.57) and gender (Pillai's Trace = 0.47, *F*(9, 93) = 9.13, *p* < .001, *η*_*p*_^*2*^ = 0.47) main effects. These effects were qualified by significant multivariate time × group (Pillais' Trace = 0.33, *F*(18, 84) = 2.29, *p* = .006, *η*_*p*_^*2*^ = 0.33) and time × gender (Pillais' Trace = 0.32, *F*(18, 84) = 2.22, *p* = .008, *η*_*p*_^*2*^ = 0.32) interactions. The results of the univariate follow-up analyses for the above mentioned multivariate effects are presented below. Full tables for each univariate follow-up analysis are provided in the online supplemental materials. Only significant effects that are not qualified by higher order effects are reported below.

#### 3.4.1 The Relationship Obsessive–Compulsive Inventory (ROCI)

**Fig. 4:**
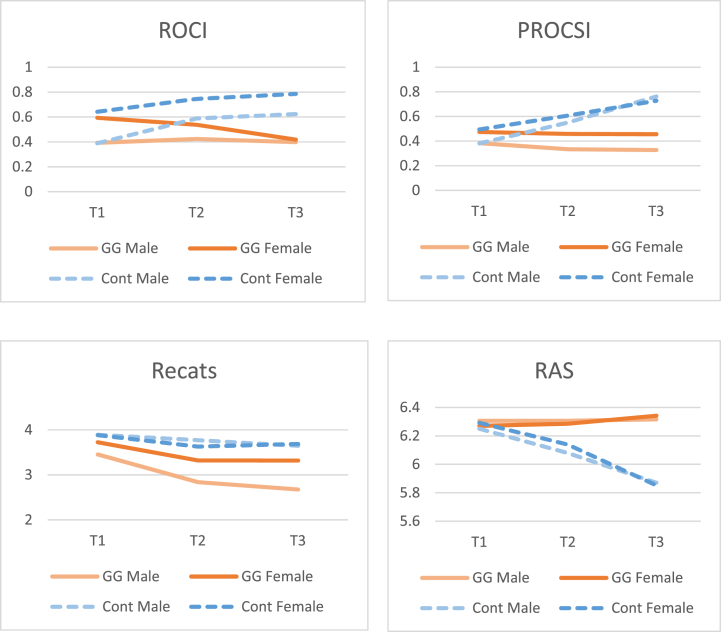

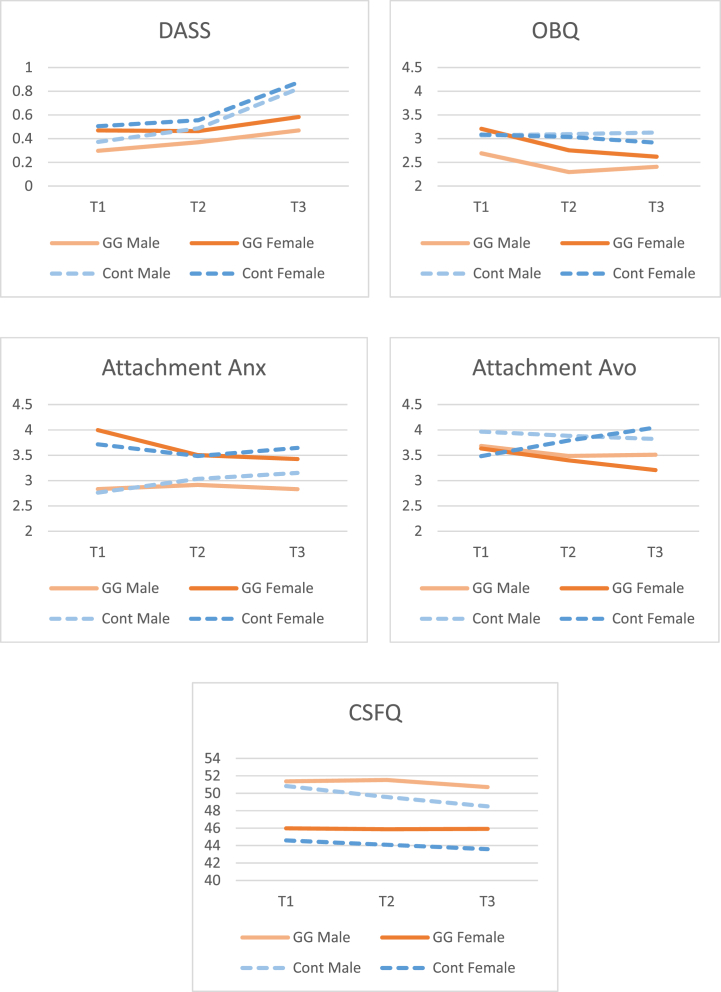
Questionnaire scores for experimental and control groups at T1, T2 and T3.

As expected, a statistically significant univariate time × group interaction effect was found (*F*(2, 202) = 5.17, *p* = .006, *η*_*p*_^*2*^ = 0.05). Bonferroni post-hoc comparisons revealed that in the control group, T2 ROCI scores were significantly higher than the scores at T1, but did not significantly differ from those at T3 (see Table 5). Conversely, in the GGRO group, no significant differences between ROCI scores were found across measurement times. In addition, a significant univariate main effect of gender was found (*F*(1, 101) = 8.42, *p* = .005, *η*_*p*_^*2*^ = 0.08), indicating that, overall, women showed higher relationship-centered ROCD levels than their male partners (see Fig. 4). No other significant effects were found.

**Table 5:** Comparisons between assessments for experimental and control groups.

	T1 *M (SD)*		T2 *M (SD)*		T3 *M (SD)*		Bonferroni post-hoc	
	Experimental	Control	Experimental	Control	Experimental	Control	Experimental	Control
ROCI	.49 (.53)	.52 (.51)	.48 (.59)	.67 (.77)	.41 (.49)	.70 (.66)	T1 *vs* T2 = *p* = 1.00 T1 *vs* T3 = *p* = .59 T2 *vs* T3 = *p* = .59	T1 *vs* T2 = *p* = .048 T1 *vs* T3 = *p* = .009 T2 *vs* T3 = *p* = 1.00
PROCSI	.43 (.44)	.44 (.40)	.40 (.45)	.58 (.60)	.39 (.44)	.75 (.66)	T1 *vs* T2 = *p* = 1.00 T1 *vs* T3 = *p* = 1.00 T2 *vs* T3 = *p* = 1.00	T1 *vs* T2 = *p* = .036 T1 *vs* T3 = *p* = .000 T2 *vs* T3 = *p* = .000
ReCats	3.59 (1.14)	3.89 (1.09)	3.08 (1.12)	3.70 (1.14)	3.00 (1.05)	3.67 (1.06)	T1 *vs* T2 = *p* = .000 T1 *vs* T3 = *p* = .000 T2 *vs* T3 = *p* = 1.00	T1 *vs* T2 = *p* = .10 T1 *vs* T3 = *p* = .08 T2 *vs* T3 = *p* = 1.00
OBQ-20	2.95 (.93)	3.08 (.88)	2.52 (.92)	3.07 (.96)	2.51 (.93)	3.02 (.89)	T1 *vs* T2 = *p* = .000 T1 *vs* T3 = *p* = .000 T2 *vs* T3 = *p* = 1.00	T1 *vs* T2 = *p* = 1.00 T1 *vs* T3 = *p* = 1.00 T2 *vs* T3 = *p* = 1.00
ECR-S- anxiety	3.42 (1.48)	3.24 (1.43)	3.21 (1.50)	3.26 (1.38)	3.13 (1.45)	3.40 (1.39)	T1 *vs* T2 = *p* = .31 T1 *vs* T3 = *p* = .04 T2 *vs* T3 = *p* = 1.00	T1 *vs* T2 = *p* = 1.00 T1 *vs* T3 = *p* = .45 T2 *vs* T3 = *p* = .57
ECR-S- avoidance	3.66 (1.13)	3.72 (1.25)	3.44 (1.21)	3.84 (1.29)	3.36 (1.35)	3.93 (1.28)	T1 *vs* T2 = *p* = .13 T1 *vs* T3 = *p* = .06 T2 *vs* T3 = *p* = 1.00	T1 *vs* T2 = *p* = .78 T1 *vs* T3 = *p* = .26 T2 *vs* T3 = *p* = 1.00
RAS	6.29 (.73)	6.27 (.63)	6.30 (.64)	6.11 (.81)	6.33 (.69)	5.86 (.97)	T1 *vs* T2 = *p* = 1.00 T1 *vs* T3 = *p* = 1.00 T2 *vs* T3 = *p* = 1.00	T1 *vs* T2 = *p* = .03 T1 *vs* T3 = *p* = .000 T2 *vs* T3 = *p* = .01

Note: *ECR-S: short form of Experience in close relationship scale; OBQ-20: short form of Obsessional Beliefs Questionnaire; RAS: Relationship assessment scale; ReCats: Relationship Catastrophization Scale; ROCI: Relationship Obsessive–Compulsive Inventory; PROCSI: Partner-Related Obsessive–Compulsive Symptoms Inventory. M: mean, SD: standard deviation*.

#### 3.4.2 Partner-Related Obsessive–Compulsive Symptoms Inventory (PROCSI)

Mauchly's Test of Sphericity indicated that the assumption of sphericity had been violated for the time variable (*χ*^*2*^(2) = 34.27, *p* < .001). Therefore, we examined the univariate main effect of time on PROCSI scores using the Greenhouse-Geisser correction. Significant main effects for time (*F*(1.55, 156.57) = 5.56, *p* = .009, *η*_*p*_^*2*^ = 0.05) and group (*F*(1, 101) = 7.28, *p* = .008, *η*_*p*_^*2*^ = 0.07) were found, but these effects were qualified by a significant time × group interaction (*F*(1.55, 156.57) = 8.91, *p* = .001, *η*_*p*_^*2*^ = 0.08). Bonferroni post-hoc comparisons showed that in the control group there was a significant increase in PROCSI scores from T1 to T2 and from T2 to T3 (see Table 5). However, the PROCSI scores of the experimental group did not differ significantly across measurement times. No other significant effects were found.

#### 3.4.3 The Relationship Catastrophization Scale (ReCats)

The findings revealed significant univariate main effects for time (*F*(2, 202) = 22.91, *p* < .001, *η*_*p*_^*2*^ = 0.19) and group (*F*(1, 101) = 12.02, *p* = .001, *η*_*p*_^*2*^ = 0.11), but these effects were qualified by the expected significant time × group interaction effect (*F*(2, 202) = 4.84, *p* = .009, *η*_*p*_^*2*^ = 0.05). Bonferroni post-hoc comparisons showed that in the experimental group, T2 ReCats scores were significantly lower than the scores at T1, but did not significantly differ from the scores at T3 (see Table 5). The ReCats scores of the control group did not differ significantly across measurement times.

#### 3.4.4 The Obsessional Beliefs Questionnaire-20 items (OBQ-20)

The results showed significant univariate time (*F*(2, 202) = 10.96, *p* < .001, *η*_*p*_^*2*^ = 0.10) and group (*F*(1, 101) = 11.91, *p* = .001, *η*_*p*_^*2*^ = 0.11) main effects, but these were qualified by the expected significant time × group interaction effect (*F*(2, 202) = 7.96, *p* < .001, *η*_*p*_^*2*^ = 0.07). Bonferroni post-hoc comparisons revealed that in the experimental group, T2 OBQ-20 scores were significantly lower than the scores at T1, but did not differ significantly from scores at T3 (see Table 5). The OBQ-20 scores of the control group did not differ significantly across measurement times. The results also revealed a significant time × gender (*F*(2, 202) = 3.41, *p* = .04, *η*_*p*_^*2*^ = 0.03) interaction. Bonferroni post-hoc comparisons showed that men showed significantly lower OBQ-20 scores at T2 (*M* = 2.72, *SD* = 0.94) as compared to T1 (*M* = 2.89, *SD* = 0.87; *p* = .04), but the scores at T3 (*M* = 2.79, *SD* = 0.98) did not significantly differ from those at T2 (*p* = .91). Women showed a similar pattern, but the decrease in OBQ-20 scores from T1 to T2 was greater for them (T1: *M* = 3.14, *SD* = 0.92; T2: *M* = 2.90, *SD* = 1.00; T3: *M* = 2.77, *SD* = 0.90; T1 *vs* T2 = *p* = .01, T2 *vs* T3 = *p* = .30).

#### 3.4.5 The depression scales (DASS-7)

Mauchly's Test of Sphericity indicated that the assumption of sphericity had been violated for the time variable (*χ*^*2*^(2) = 37.03, *p* < .001). To examine the univariate main effect of time on DASS-7 scores, the Greenhouse-Geisser correction was used. The results showed a significant time main effect (*F*(1.53, 154.26) = 14.01, *p* < .001, *η*_*p*_^*2*^ = 0.12), suggesting that there was a significant change in DASS-7 scores across measurement times. Additionally, a significant gender main effect (*F*(1, 101) = 5.71, *p* = .02, *η*_*p*_^*2*^ = 0.05) and a marginal group main effect (*F*(1, 101) = 4.06, *p* = .047, *η*_*p*_^*2*^ = 0.04) were revealed, indicating that women had more depressive symptoms than men, and that, overall, couples in the experimental group had marginally lower levels of depressive symptoms as compared to the control group (see Fig. 4). There were no other significant effects found.

#### 3.4.6 The short version of the experiences in close Relationships scale (ECR-S)

##### 3.4.6.1 ECR-S anxiety

As expected, a statistically significant univariate time × group interaction was found (*F*(2, 202) = 3.82, *p* = .02, *η*_*p*_^*2*^ = 0.04). The findings of Bonferroni post-hoc comparisons revealed that in the experimental group, T2 ECR-S anxiety scores did not significantly differ from those at T1 and T3, but T3 scores were significantly lower than the scores at T1 (see Table 5). There was no significant difference between ECR-S anxiety scores in the control group across measurement times. In addition, a significant time × gender interaction was found (*F*(2, 202) = 6.48, *p* = .002, *η*_*p*_^*2*^ = 0.06). The Bonferroni post-hoc comparisons showed that the ECR-S anxiety scores of women at T2 (*M* = 3.49, *SD* = 1.46) were significantly lower than at T1 (*M* = 3.85, *SD* = 1.42, *p* = .01), but did not significantly differ from the scores at T3 (*M* = 3.54, *SD* = 1.39; *p* = 1.00). Men did not have a significant difference in ECR-S anxiety scores across measurement times (T1: *M* = 2.80, *SD* = 1.29; T2: *M* = 2.97, *SD* = 1.37; T3: *M* = 2.99, *SD* = 1.41; T1 *vs* T2 = *p* = .34, T2 *vs* T3 = *p* = 1.00). No other significant effects were found.

##### 3.4.6.2 ECR-S avoidance

Mauchly's Test of Sphericity revealed that the assumption of sphericity had been violated for the time variable (*χ*^*2*^(2) = 6.62, *p* = .04). To examine the univariate main effect of time on ECR-S avoidance scores, the Greenhouse-Geisser correction was employed. A significant group main effect was revealed (*F*(1, 101) = 5.64, *p* = .02, *η*_*p*_^*2*^ = 0.05). This effect was qualified by the expected significant time × group interaction effect (*F*(1.88, 189.84) = 5.33, *p* = .007, *η*_*p*_^*2*^ = 0.05), however no statistically significant comparisons were found in the Bonferroni post-hoc test (see Table 5).

#### 3.4.7 The short version of the relationship assessment scale (RAS)

The results indicated that the assumption of Mauchly's sphericity was not met for the time variable (*χ*^*2*^ (2) = 21.03, *p* < .001). Therefore, the Greenhouse-Geisser correction was used to analyze the main effect of time on RAS scores. The findings revealed significant univariate main effects for time (*F*(1.68, 169.80) = 5.17, *p* = .01, *η*_*p*_^*2*^ = 0.05) and group (*F*(1, 101) = 4.55, *p* = .04, *η*_*p*_^*2*^ = 0.04), but these effects were qualified by the expected significant time × group interaction effect (*F*(1.68, 169.80) = 7.80, *p* = .001, *η*_*p*_^*2*^ = 0.07). The Bonferroni post-hoc comparisons showed that in the control group, T2 scores were significantly lower than the scores at the T1 and significantly higher than those at T3 (see Table 5). There was no significant difference between RAS scores in the experimental group across measurement times. No other significant effects were found.

#### 3.4.8 The Changes in Sexual Functioning Questionnaire-14 (CSFQ-14)

Mauchly's Test of Sphericity indicated that the assumption of sphericity had been violated for the time variable (*χ*^*2*^ (2) = 24.59, *p* < .001). To examine the main effect of time on CSFQ-14 scores, the Greenhouse-Geisser correction was used. The results showed significant univariate time (*F*(1.64, 165.85) = 3.63, *p* = .04, *η*_*p*_^*2*^ = 0.04) and gender (*F*(1, 101) = 59.63, *p* < .001, *η*_*p*_^*2*^ = 0.37) main effects on CSFQ-14 scores. These results indicate that there was a significant change in CSFQ-14 scores across measurement times, and that men had better sexual function than women (see Fig. 4). No other statistically significant effects were found.

#### 3.4.9 Resilience task

A 2 (Group: experimental, control) × 2 (Gender: male, female) between-within analysis of variance (ANOVA) was performed to compare current relationship-centered ROCD symptoms between the experimental and control groups. As expected, a significant group main effect was found (*F*(1, 101) = 14.89, *p* < .001, *η*_*p*_^*2*^ = 0.13), suggesting that the experimental group showed significantly more resilience to relationship-centered ROCD triggering cues (*M* = 3.29, *SD* = 1.56) compared to the control group (*M* = 4.23, *SD* = 1.70). In addition, a statistically significant gender main effect was revealed (*F*(1, 101) = 16.70, *p* < .001, *η*_*p*_^*2*^ = 0.14), indicating that men were significantly more resilient to these relationship-centered ROCD triggering cues (*M* = 3.38, *SD* = 1.58) compared to women (*M* = 4.18, *SD* = 1.73). An interaction effect was not found (*F*(1, 101) = 2.07, *p* = .15, *η*_*p*_^*2*^ = 0.02).

## 4 Discussion

Healthy romantic relationships have been shown to conserve mental health and wellbeing during challenging times. Pre-existing vulnerabilities such as ROCD symptoms, however, may make it difficult to maintain healthy relationships. The current study serves to illustrate the effectiveness of a scalable, low-cost mobile intervention in increasing resilience to ROCD symptoms, cognition and related triggers by challenging OCD and ROCD-related maladaptive beliefs.

Consistent with our hypotheses, although couples in the control group of our study showed a significant increase in ROCD symptoms during the study period, GGRO users did not report such an increase, neither immediately following app use nor at follow-up. Also, compared with the control group, GGRO users in our study showed significant reductions in OCD and ROCD cognitions. Moreover, large effect-size differences between GGRO users and the control group were found following the resilience task indicating stronger resilience in the GGRO group.

Previous RCTs have linked app use with reduction in R/OCD symptoms and associated maladaptive beliefs. Replicating previous studies, our findings indicated significant reductions in maladaptive beliefs associated with R/OCD symptoms [45,47]. In this study, however, GGRO use was not associated with symptom reduction, but rather protected against the increase in symptoms seen in the control group.

The observed increase in R/OCD symptoms in the control group of our RCT may be due to various stress-inducing environmental factors. However, our results are congruent with the predictions of CBT models of R/OCD [8,9,27,28] pertaining to individuals with relatively low baseline R/OCD symptom levels. According to these models, ongoing catastrophic appraisals of common intrusions lead to counterproductive efforts (e.g., compulsive behaviors) intended to alleviate the distress associated with these intrusions. Reductions in maladaptive beliefs that fuel these appraisals would therefore prevent an increase in R/OCD symptoms during stressful periods when relationship-related intrusions might increase [24]. This should be particularly evident in community cohorts whereby the OCD cycle does yet perpetuate itself.

The results from our resilience task also lend support to the above-mentioned resilience hypothesis. We found large effect-size differences between GGRO users and the control group in the resilience task indicating couples using the app were less sensitive to ROCD-related triggers. This finding aligns with a previous RCT that employed the GGtude platform [43]. The results from this RCT suggested that targeting beliefs associated with body image distress (e.g., exaggerated importance of appearance) increased resilience to body image triggers such as exposure to an Instagram account specifically created to trigger body image distress.

Although participants in the control group of our study showed a decrease in relationship satisfaction during the study period, users of the app did not show such a decrease during the study and at follow-up. Indeed, previous findings indicate strong correlations between ROCD symptoms and relationship dissatisfaction [9,12]. Our finding that both ROCD symptoms and relationship dissatisfaction increase in the control group but not in the app use group is therefore not surprising.

We found that app use was associated with lower anxious attachment orientation over time. The lowering of anxious attachment orientation, however, was only evident at the 1-month follow-up. This might suggest that app use has a lingering effect on this attachment orientation. Indeed, reductions in attachment insecurity several months following termination of traditional CBT have been previously reported [[71], [72], [73]] supporting the proposal that app use may reduce long-term vulnerability to ROCD symptoms.

Doron et al. [9] showed that the link between ROCD symptoms and decreased sexual satisfaction was mediated by relationship satisfaction. They suggested that reducing ROCD symptoms may improve the sexual experience. This suggestion, however, was not supported by our findings. GGRO does not target maladaptive beliefs underlying sexual dysfunction. Future research may examine whether targeting such beliefs would be more effective for achieving improvement in couples' sexual experiences.

Consistent with previous studies using GGRO [16,47], no statistically significant changes in depression levels were observed between groups across measurement times. The lack of reduction in depression symptoms in the current sample may be due to our sample's relatively low initial depression levels. Alternatively, modules specifically targeting maladaptive beliefs associated with depression for longer periods of time (e.g. Ref. [44]) may be more effective in reducing depression symptoms in couples.

### 4.1 Strengths, limitations, and future directions

In our study a noteworthy dropout rate was detected (22.33 %). Nevertheless, such dropout rates are not uncommon in studies evaluating the effectiveness of mobile applications for mental health purposes. In fact, a recent meta-analysis indicated that the mean dropout rates for mHealth apps is 33.2 % when accounting for publication bias and 24.1 % without accounting for such bias [74].

Our study included a non-clinical community sample of couples with relatively low baseline ROCD symptoms levels. Although recent reviews support the inclusion of nonclinical individuals in OCD-related research (e.g. Ref. [75]), it's important to acknowledge that clinical populations may differ from nonclinical participants in terms of symptom-related impairment. In future studies it may be advantageous to explore the efficacy of such an intervention among individuals with diagnosed OCD. Finally, our study used a waitlist control group. Future studies would benefit from including an active control group using a similar apps targeting beliefs unrelated to ROCD.

Despite the above-mentioned limitations and pending replication of the findings, the findings of our study carry significant theoretical and practical implications. To our knowledge, this is the first study to show the beneficial effects of app use by both partners on relationship related variables and resilience. Couples using the app showed less maladaptive beliefs related to relationships and less attachment anxiety (at follow up). Couples using the app also showed resilience to relationship dissatisfaction and relationship obsessions. App use by both partners may benefit both partners of a romantic dyad and the relationship itself.

CBT models of psychopathology suggest that maladaptive beliefs play a pivotal role in the negative (and often catastrophic) misinterpretation of commonly occurring events or cognitions resulting in psychopathology [76]. Our finding that targeting such beliefs increases resilience to psychopathology in both members of the romantic dyad lends further support to these models. Indeed, previous studies using the GGtude platforms consistently show the benefits of targeting maladaptive beliefs associated with various psychopathologies [44,45,[47], [48], [49], [50], [51],^77^].

Our results indicate that purely cognitive exercises may increase resilience and reduce maladaptive beliefs, which has theoretical and practical implications. In face of recent challenges [78], our findings give empirical support to the efficacy of purely cognitive interventions for OCD and psychopathology. In practical terms, purely cognitive interventions can be relatively brief, require less offline efforts (e.g., in-vivo exposures), may be less intimidating to users than behavioral interventions, and are very flexible and easily integrated in mHealth application, making them attractive to a wide variety of users.

The mHealth intervention assessed in this study was used as a stand-alone intervention. Nevertheless, similar mHealth interventions could be used in conjunction with face-to-face therapy. For instance, our study group is currently assessing a face-to-face protocol that incorporates app use between sessions. The integration of an mHealth app in this case is believed to work in synergy with face-to-face treatment, enhance the effects of therapy and is also used as a relapse prevention tool.

### 4.2 Conclusions

Considering the above-mentioned limitations, and pending further replication of our findings, our results suggest that concurrent use of brief, daily cognitive exercises by both romantic partners may significantly increase resilience to ROCD triggers and decrease vulnerability and maintaining factors of ROCD and related relational phenomena.

## Data availability statement

Data will be made available on request.

## CRediT authorship contribution statement

**Milana Gorelik:** Writing – review & editing, Writing – original draft, Validation, Software, Resources, Project administration, Methodology, Investigation, Formal analysis, Data curation, Conceptualization. **Ohad Szepsenwol:** Writing – review & editing, Writing – original draft, Validation, Methodology, Formal analysis, Data curation. **Guy Doron:** Writing – review & editing, Writing – original draft, Supervision, Software, Resources, Methodology, Formal analysis, Conceptualization.

## Declaration of competing interest

The authors declare the following financial interests/personal relationships which may be considered as potential competing interestsMilana Gorelik declares that she has no conflict of interestOhad Szepsenwol declares that she has no conflict of interest.Guy Doron is one of the authors of the paper and a co-developer of GGRO. Guy Doron is also a co-founder of GGtude Ltd. GGRO is the subject of this evaluation and therefore has financial interest to GGtude Ltd.

## References

[1] Braithwaite S., Holt-Lunstad J. (2017). Romantic relationships and mental health. Curr Opin Psychol.

[2] Loving T.J., Slatcher R.B., Simpson J.A., Campbell L. (2013). The Oxford Handbook of Close Relationships.

[3] Kansky J. (2018).

[4] Luciano E.C., Orth U. (2017). Transitions in romantic relationships and development of self-esteem. J. Pers. Soc. Psychol..

[5] Shaver P.R., Mikulincer M., Mikulincer M., Shaver P.R. (2014). Mechanisms of Social Connection: from Brain to Group.

[6] Pietromonaco P.R., Collins N.L. (2017). Interpersonal mechanisms linking close relationships to health. Am. Psychol..

[7] Pietromonaco P.R., Overall N.C. (2021). Applying relationship science to evaluate how the COVID-19 pandemic may impact couples' relationships. Am. Psychol..

[8] Doron G., Derby D.S., Szepsenwol O. (2014). Relationship obsessive-compulsive disorder (ROCD): a conceptual framework. J Obsess-Compuls Relat Disord..

[9] Doron G., Mizrahi M., Szepsenwol O., Derby D. (2014). Right or flawed: relationship obsessions and sexual satisfaction. J. Sex. Med..

[10] Trak E., Inozu M. (2019). Developmental and self-related vulnerability factors in relationship-centered obsessive compulsive disorder symptoms: a moderated mediation model. J Obsess-Compuls Relat Disord..

[11] Szepsenwol O., Shahar B., Doron G. (2016). Letting it linger: exploring the longitudinal effects of relationship-related obsessive-compulsive phenomena. J Obsess-Compuls Relat Disord..

[12] Doron G., Derby D.S., Szepsenwol O., Talmor D. (2012). Tainted love: exploring relationship-centered obsessive compulsive symptoms in two non-clinical cohorts. J Obsess-Compuls Relat Disord..

[13] Brandes O., Stern A., Doron G. (2020). I just can't trust my partner”: Evaluating associations between untrustworthiness obsessions, relationship obsessions and couples’ violence. J Obsess-Compuls Relat Disord.

[14] Doron G., Derby D.S., Szepsenwol O., Talmor D. (2012). Flaws and all: exploring partner-focused obsessive-compulsive symptoms. J Obsess-Compuls Relat Disord..

[15] Fernandez S., Sevil C., Moulding R. (2021). Feared self and dimensions of obsessive compulsive symptoms: sexual orientation-obsessions, relationship obsessions, and general OCD symptoms. J Obsess-Compuls Relat Disord..

[16] Roncero M., Belloch A., Doron G. (2018). A novel approach to challenging OCD related beliefs using a mobile-app: an exploratory study. J. Behav. Ther. Exp. Psychiatr..

[17] Melli G., Bulli F., Doron G., Carraresi C. (2018). Maladaptive beliefs in relationship obsessive compulsive disorder (ROCD): replication and extension in a clinical sample. J Obsess-Compuls Relat Disord..

[18] Tinella L., Lunardi L., Rigobello L., Bosco A., Mancini F. (2023). Relationship Obsessive Compulsive Disorder (R-OCD): the role of relationship duration, fear of guilt, and personality traits. J Obsess-Compuls Relat Disord..

[19] Toroslu B., Çırakoğlu O.C. (2022). Do perfectionism and intolerance of uncertainty mediate the relationship between early maladaptive schemas and relationship and partner related obsessive–compulsive symptoms?. Curr. Psychol..

[20] Ghomian S., Shaeiri M.R., Farahani H. (2021). Relationship obsessive compulsive disorder (ROCD) in Iranian culture: symptoms, causes and consequences. J Fundam Ment Health.

[21] Levy A., Tibi L., Szepsenwol O., Doron G. (2020). Why do I obsess about my child's flaws?”: Assessing the role of parental self‐vulnerabilities in parent–child relationship obsessive compulsive disorder (ROCD) symptoms. Clin Psychol.

[22] Ratzoni N., Doron G., Frenkel T.I. (2021). Initial evidence for Symptoms of Postpartum Parent-Infant Relationship Obsessive Compulsive Disorder (PI-ROCD) and associated risk for perturbed maternal behavior and infant social disengagement from mother. Front. Psychiatr..

[23] Littman R., Leibovits G., Halfon C.N., Schonbach M., Doron G. (2023). Interpersonal transmission of ROCD symptoms and susceptibility to infidelity in romantic relationships. J Obsessive-Compuls Relat Disord..

[24] Doron G., Derby D. (2017). Assessment and treatment of relationship‐related OCD symptoms (ROCD) A modular approach. The Wiley Handbook of Obsessive Compulsive Disorders.

[25] Doron G., Derby D., Szepsenwol O., Nahaloni E., Moulding R. (2016). Relationship obsessive–compulsive disorder: interference, symptoms, and maladaptive beliefs. Front. Psychiatr..

[26] Taylor S., Abramowitz J.S., McKay D. (2007). Psychological Treatment of Obsessive-Compulsive Disorder: Fundamentals and beyond.

[27] Obsessive Compulsive Cognitions Working Group (1997). Cognitive assessment of obsessive-compulsive disorder. Behav. Res. Ther..

[28] Obsessive Compulsive Cognitions Working Group (2005). Psychometric validation of the obsessive belief questionnaire and interpretation of intrusions inventory—Part 2: factor analyses and testing of a brief version. Behav. Res. Ther..

[29] Rachman S. (1998). A cognitive theory of obsessions: elaborations. Behav. Res. Ther..

[30] Salkovskis P.M. (1985). Obsessional-compulsive problems: a cognitive-behavioural analysis. Behav. Res. Ther..

[31] Doron G., Szepsenwol O., Karp E., Gal N. (2013). Obsessing about intimate relationships: testing the double relationship vulnerability hypothesis. J. Behav. Ther. Exp. Psychiatr..

[32] Melli G, Caccico L, Micheli L, Bulli F, Doron G. Pathological narcissism and relationship obsessive-compulsive disorder (ROCD) symptoms: exploring the role of vulnerable narcissism J. Clin. Psychol.. .. 10.1002/jclp.23601 37830404

[33] Doron G., Szepsenwol O. (2015). Partner-focused obsessions and self-esteem: an experimental investigation. J. Behav. Ther. Exp. Psychiatr..

[34] McKay D., Sookman D., Neziroglu F., Wilhelm S., Stein D.J., Kyrios M. (2015). Efficacy of cognitive-behavioral therapy for obsessive-compulsive disorder. Psychiatr. Res..

[35] National Collaborating Centre for Mental Health (UK) (2006).

[36] Abramowitz J.S. (2006).

[37] Foa E.B. (2010). Cognitive behavioral therapy of obsessive-compulsive disorder. Dialogues Clin. Neurosci..

[38] Marques L., LeBlanc N.J., Weingarden H.M., Timpano K.R., Jenike M., Wilhelm S. (2010). Barriers to treatment and service utilization in an internet sample of individuals with obsessive–compulsive symptoms. Depress. Anxiety.

[39] O'Neill J., Feusner J.D. (2015). Cognitive-behavioral therapy for obsessive–compulsive disorder: access to treatment, prediction of long-term outcome with neuroimaging. Psychol. Res. Behav. Manag..

[40] Mahoney A.E., Mackenzie A., Williams A.D., Smith J., Andrews G. (2014). Internet cognitive behavioural treatment for obsessive compulsive disorder: a randomised controlled trial. Behav. Res. Ther..

[41] Van Ameringen M., Turna J., Khalesi Z., Pullia K., Patterson B. (2017). There is an app for that! the current state of mobile applications (apps) for DSM‐5 obsessive‐compulsive disorder, posttraumatic stress disorder, anxiety and mood disorders. Depress. Anxiety.

[42] Gamoran A., Doron G. (2023). Effectiveness of brief daily training using a mobile app in reducing obsessive compulsive disorder (OCD) symptoms: examining real world data of “OCD.app - anxiety, mood & sleep”. J Obsess-Compuls Relat Disord..

[43] Aboody D., Siev J., Doron G. (2020). Building resilience to body image triggers using brief cognitive training on a mobile application: a randomized controlled trial. Behav. Res. Ther..

[44] Ben-Zeev D., Chander A., Tauscher J., Buck B., Nepal S., Campbell A. (2021). A smartphone intervention for people with serious mental illness: fully remote randomized controlled trial of CORE. J. Med. Internet Res..

[45] Cerea S., Ghisi M., Bottesi G., Carraro E., Broggio D., Doron G. (2020). Reaching reliable change using short, daily, cognitive training exercises delivered on a mobile application: the case of Relationship Obsessive Compulsive Disorder (ROCD) symptoms and cognitions in a subclinical cohort. J. Affect. Disord..

[46] Giraldo‐O'Meara M., Doron G. (2021 May 4). Can self-esteem be improved using short daily training on mobile applications? Examining real world data of GG Self-esteem users. Clin. Psychol..

[47] Roncero M., Belloch A., Doron G. (2019). Can brief, daily training using a mobile app help change maladaptive beliefs? Crossover randomized controlled trial. JMIR mHealth uHealth.

[48] Akin-Sari B., Inozu M., Haciomeroglu A.B., Cekci B.C., Uzumcu E., Doron G. (2022 Sep 1). Cognitive training via a mobile application to reduce obsessive-compulsive-related distress and cognitions during the COVID-19 outbreaks: a randomized controlled trial using a subclinical cohort. Behav. Ther..

[49] Akin-Sari B., Inozu M., Haciomeroglu A.B., Trak E., Tufan D., Doron G. (2022). Cognitive training using a mobile app as a coping tool against COVID-19 distress: a crossover randomized controlled trial. J. Affect. Disord..

[50] Cerea S., Doron G., Manoli T., Patania F., Bottesi G., Ghisi M. (2022). Cognitive training via a mobile application to reduce some forms of body dissatisfaction in young females at high-risk for body image disorders: a randomized controlled trial. Body Image.

[51] Abramovitch A., Uwadiale A., Robinson A. (2023). A randomized clinical trial of a gamified app for the treatment of perfectionism. Br. J. Clin. Psychol..

[52] Brewin C.R. (2006). Understanding cognitive behaviour therapy: a retrieval competition account. Behav. Res. Ther..

[53] Balcetis E., Cole S. (2009). Body in mind: the role of embodied cognition in self‐regulation. Soc Personal Psychol Compass.

[54] Gardner B., Lally P., Wardle J. (2012). Making health habitual: the psychology of 'habit-formation' and general practice. Br. J. Gen. Pract..

[55] Jacobson N.S., Truax P., Kazdin A.E. (1992). Methodological Issues & Strategies in Clinical Research.

[56] Melli G., Carraresi C., Pinto A., Caccico L., Micheli E. (2018). Valutare il disturbo ossessivo-compulsivo da relazione: proprietà psicometriche della versione italiana di ROCI e PROCSI. Psicoter. Cogn. Comportamentale.

[57] Faul F., Erdfelder E., Buchner A., Lang A.-G. (2009). Statistical power analyses using G*Power 3.1: tests for correlation and regression analyses. Behav. Res. Methods.

[58] Hendrick S.S., Dicke A., Hendrick C. (1998). The relationship assessment scale. J. Soc. Pers. Relat..

[59] Vaughn M.J., Matyastik Baier M.E. (1999). Reliability and validity of the relationship assessment scale. Am. J. Fam. Ther..

[60] Keller A., McGarvey E.L., Clayton A.H. (2006). Reliability and construct validity of the changes in sexual functioning questionnaire short-form (CSFQ-14). J. Sex Marital Ther..

[61] Clayton A.H., McGarvey E.L., Clavet G.J., Piazza L. (1997). Comparison of sexual functional in clinical and nonclinical populations using the changes in sexual functioning questionnaire (CSFQ). Psychopharmacol. Bull..

[62] Brennan K.A., Clark C.L., Shaver P.R. (1998). Self-report measurement of adult attachment: an integrative overview. Attachment Theory and Close Relationships.

[63] Wei M., Russell D.W., Mallinckrodt B., Vogel D.L. (2007). The experiences in close relationship scale (ECR)-short form: reliability, validity, and factor structure. J. Pers. Assess..

[64] Moulding R., Anglim J., Nedeljkovic M., Doron G., Kyrios M., Ayalon A. (2011). The obsessive beliefs questionnaire (OBQ): examination in nonclinical samples and development of a short version. Assessment.

[65] Lovibond P.F., Lovibond S.H. (1995). The structure of negative emotional states: comparison of the depression anxiety stress scales (DASS) with the beck depression and anxiety inventories. Behav. Res. Ther..

[66] Gupta S.K. (2011). Intention-to-treat concept: a review. Perspect Clin Res.

[67] Van Buuren S., Groothuis-Oudshoorn K. (2011). mice: multivariate imputation by chained equations in R. J. Stat. Software.

[68] Lall R. (2016). How multiple imputation makes a difference. Polit. Anal..

[69] Benjamini Y., Hochberg Y. (1995). Controlling the false discovery rate: a practical and powerful approach to multiple testing. J R Stat Soc Series B Methodol.

[70] Keselman H.J., Cribbie R., Holland B. (1999). The pairwise multiple comparison multiplicity problem: an alternative approach to familywise and comparison wise Type I error control. Psychol. Methods.

[71] Lange J., Goerigk S., Nowak K., Rosner R., Erhardt A. (2021). Attachment style change and working alliance in panic disorder patients treated with cognitive behavioral therapy. Psychotherapy.

[72] Strauß B., Altmann U., Manes S., Tholl A., Koranyi S., Nolte T., Beutel M.E., Wiltink J., Herpertz S., Hiller W., Hoyer J. (2018). Changes of attachment characteristics during psychotherapy of patients with social anxiety disorder: results from the SOPHO-Net trial. PLoS One.

[73] Zalaznik D., Strauss A.Y., Halaj A., Fradkin I., Ebert D.D., Andersson G., Huppert J.D. (2021). Anxious attachment improves and is predicted by anxiety sensitivity in internet-based, guided self-help cognitive behavioral treatment for panic disorder. J. Counsel. Psychol..

[74] Linardon J., Fuller-Tyszkiewicz M. (2020). Attrition and adherence in smartphone-delivered interventions for mental health problems: a systematic and meta-analytic review. J. Consult. Clin. Psychol..

[75] Abramowitz J.S., Fabricant L.E., Taylor S., Deacon B.J., McKay D., Storch E.A. (2014). The relevance of analogue studies for understanding obsessions and compulsions. Clin. Psychol. Rev..

[76] Beck A.T., Dozois D.J. (2011). Cognitive therapy: current status and future directions. Annu. Rev. Med..

[77] Cerea S., Ghisi M., Bottesi G., Manoli T., Carraro E., Doron G. (2021). Cognitive behavioral training using a mobile application reduces body image-related symptoms in high-risk female university students: a randomized controlled study. Behav. Ther..

[78] Anholt G.E., Kalanthroff E. (2014). Do we need a cognitive theory for obsessive-compulsive disorder?. Clin Neuropsychiatry.

