# Supplementary material for: Promoting couples’ resilience to relationship obsessive compulsive disorder (ROCD) symptoms using a CBT-based mobile application: A randomized controlled trial
Source: Heliyon. 2023 Oct 28;9(11):e21673. doi: 10.1016/j.heliyon.2023.e21673 (PMC10656241; doi:10.1016/j.heliyon.2023.e21673)

**The ROCI**

The following statements describe the way people may experience intimate relationships. We are interested in the way **you** experience intimate relationships. Please rate the extent to which such thoughts and behaviors describe your experiences in intimate relationships**.**

The numbers refer to the following verbal labels:

| Not at all  0 | A little  1 | Moderately  2 | A lot  3 | Very much  4 |
| --- | --- | --- | --- | --- |

| 1. | The thought that I don't really love my partner haunts me |  | 0 | 1 | 2 | 3 | 4 |
| --- | --- | --- | --- | --- | --- | --- | --- |
| 2. | I find it easy to dismiss my doubts about my partner |  | 0 | 1 | 2 | 3 | 4 |
| 3. | I constantly doubt my relationship |  | 0 | 1 | 2 | 3 | 4 |
| 4. | I find it difficult to dismiss doubts regarding my partner's love for me |  | 0 | 1 | 2 | 3 | 4 |
| 5. | I check and recheck whether my relationship feels "right" |  | 0 | 1 | 2 | 3 | 4 |
| 6. | I am constantly looking for evidence that my partner really loves me |  | 0 | 1 | 2 | 3 | 4 |
| 7. | I feel that I must remind myself over and over again why I love my partner |  | 0 | 1 | 2 | 3 | 4 |
| 8. | I am sure my partner loves me |  | 0 | 1 | 2 | 3 | 4 |
| 9. | I am extremely disturbed by thoughts that something is "not right" in my relationship |  | 0 | 1 | 2 | 3 | 4 |
| 10. | I continuously doubt my love for my partner |  | 0 | 1 | 2 | 3 | 4 |
| 11. | I keep asking my partner whether she/he really loves me |  | 0 | 1 | 2 | 3 | 4 |
| 12. | I frequently seek reassurance that my relationship is "right" |  | 0 | 1 | 2 | 3 | 4 |
| 13. | I am constantly bothered by the thought that my partner doesn't really want to be with me |  | 0 | 1 | 2 | 3 | 4 |
| 14. | I feel a need to repeatedly check how much I love my partner |  | 0 | 1 | 2 | 3 | 4 |
|  |  |  |  |  |  |  |  |

**The PROCSI**

The following statements describe the way people may experience intimate relationships. We are interested in the way **you** experience intimate relationships. Please rate the extent to which such thoughts and behaviors describe your experiences in intimate relationships**.**

The numbers refer to the following verbal labels:

| Not at all  0 | A little  1 | Moderately  2 | A lot  3 | Very much  4 |
| --- | --- | --- | --- | --- |

| 1. | I am happy with my partner's morality level |  | 0 | 1 | 2 | 3 | 4 |
| --- | --- | --- | --- | --- | --- | --- | --- |
| 2. | I repeatedly evaluate my partner's social functioning |  | 0 | 1 | 2 | 3 | 4 |
| 3. | I am constantly questioning whether my partner is deep and intelligent enough |  | 0 | 1 | 2 | 3 | 4 |
| 4. | I am satisfied by with my partner's appearance |  | 0 | 1 | 2 | 3 | 4 |
| 5. | I am troubled by thoughts about my partner's social skills |  | 0 | 1 | 2 | 3 | 4 |
| 6. | I am constantly bothered by doubts about my partner's morality level |  | 0 | 1 | 2 | 3 | 4 |
| 7. | I find it hard to dismiss the thought that my partner is mentally unbalanced |  | 0 | 1 | 2 | 3 | 4 |
| 8. | I often seek reassurance (from friends, family, etc.) about whether my partner is smart enough |  | 0 | 1 | 2 | 3 | 4 |
| 9. | When I am with my partner I find it hard to ignore her physical flaws |  | 0 | 1 | 2 | 3 | 4 |
| 10. | I keep comparing my partner's ability to "achieve something" in life to that of other men/women |  | 0 | 1 | 2 | 3 | 4 |
| 11. | I can't stop comparing my partner's intelligence level to that of other men/women |  | 0 | 1 | 2 | 3 | 4 |
| 12. | I find it difficult to control my tendency to compare my partner's emotional responses to those of other men/women |  | 0 | 1 | 2 | 3 | 4 |
| 13. | The thought that my partner is not intelligent enough bothers me greatly |  | 0 | 1 | 2 | 3 | 4 |
| 14. | I am constantly bothered by thoughts regarding the flaws in my partner's physical appearance |  | 0 | 1 | 2 | 3 | 4 |
| 15. | The thought that my partner is not a "good and moral" person bothers me on a daily basis |  | 0 | 1 | 2 | 3 | 4 |
| 16. | I feel good about my partner's level of intelligence |  | 0 | 1 | 2 | 3 | 4 |
| 17. | I keep looking for evidence that my partner is moral enough |  | 0 | 1 | 2 | 3 | 4 |
| 18. | Thoughts about my partner's poor functioning in social situations bother me on a daily basis |  | 0 | 1 | 2 | 3 | 4 |
| 19. | Every time I'm reminded of my partner I think about the flaw in his/her appearance |  | 0 | 1 | 2 | 3 | 4 |
| 20. | I'm constantly examining my partner's morality level |  | 0 | 1 | 2 | 3 | 4 |
| 21. | I keep trying to compensate for my partner's social deficiencies |  | 0 | 1 | 2 | 3 | 4 |
| 22. | I am bothered by doubts about my partner's emotional stability |  | 0 | 1 | 2 | 3 | 4 |
| 23. | I am happy with my partner's social skills |  | 0 | 1 | 2 | 3 | 4 |
| 24. | I keep examining whether my partner acts in a strange manner |  | 0 | 1 | 2 | 3 | 4 |
| 25. | I am extremely preoccupied with assessing my partner's ability to "make something of himself/herself" |  | 0 | 1 | 2 | 3 | 4 |
| 26. | I feel an uncontrollable urge to compare my partner's physical flaws with those of other men/women |  | 0 | 1 | 2 | 3 | 4 |
| 27. | When I think of my partner I wonder whether he/she is the sort of person who can succeed in the modern world |  | 0 | 1 | 2 | 3 | 4 |
| 28. | I keep looking for evidence of my partner's occupational success |  | 0 | 1 | 2 | 3 | 4 |
|  |  |  |  |  |  |  |  |

RECATS

This inventory lists different attitudes or beliefs that people sometimes hold. Read each statement carefully and decide how much you agree or disagree with it. For each statement, choose the number matching the answer that best describes how you think. Because people are different, there are no right or wrong answers. To decide whether a given statement is typical of your way of looking at things, simple keep in mind what you are like most of the time. Use the following scale.

| 1  Disagree  Very Much | 2  Disagree  Moderately | 3  Disagree a little | 4  Neither agree nor disagree | 5  Agree a little | 6  Agree moderately | 7  Agree very much |
| --- | --- | --- | --- | --- | --- | --- |

Rate your replies as follows:

| 1. | I would prefer almost anything over having to deal with the consequences of breaking up with my partner | 1 | 2 | 3 | 4 | 5 | 6 | 7 |
| --- | --- | --- | --- | --- | --- | --- | --- | --- |
| 2. | I always thought that intimate relationships are stupid and it’s better to be alone | 1 | 2 | 3 | 4 | 5 | 6 | 7 |
| 3. | For me, being in an imperfect relationship is like betraying myself | 1 | 2 | 3 | 4 | 5 | 6 | 7 |
| 4. | Being without a partner would cause great pain to me and everyone around me | 1 | 2 | 3 | 4 | 5 | 6 | 7 |
| 5. | I think breaking up with a partner is one of the worst things that can happen to anyone | 1 | 2 | 3 | 4 | 5 | 6 | 7 |
| 6. | I think bad romantic decisions almost always cost dearly | 1 | 2 | 3 | 4 | 5 | 6 | 7 |
| 7. | The thought of going through life without a partner scares me to death | 1 | 2 | 3 | 4 | 5 | 6 | 7 |
| 8. | In my opinion, a romantic relationship that doesn’t always feel right is probably a destructive relationship | 1 | 2 | 3 | 4 | 5 | 6 | 7 |
| 9. | “It is not good that the man should be alone” is a verse I live my life by | 1 | 2 | 3 | 4 | 5 | 6 | 7 |
| 10. | It is clear to me that you can have a very good life even being in a bad relationship | 1 | 2 | 3 | 4 | 5 | 6 | 7 |
| 11. | I am convinced that breaking up with my partner might cause both of us irreparable damage | 1 | 2 | 3 | 4 | 5 | 6 | 7 |
| 12. | If there is something I don’t wish anyone, it is being alone in the world, without a relationship | 1 | 2 | 3 | 4 | 5 | 6 | 7 |
| 13. | As far as I am concerned, there is nothing harder than dealing with a break-up | 1 | 2 | 3 | 4 | 5 | 6 | 7 |
| 14. | I believe that making the wrong romantic choice is often a terrible thing | 1 | 2 | 3 | 4 | 5 | 6 | 7 |
| 15. | I feel I can’t take back my romantic decisions | 1 | 2 | 3 | 4 | 5 | 6 | 7 |
| 16. | I believe that being in the wrong relationship almost always leads to a wasted life | 1 | 2 | 3 | 4 | 5 | 6 | 7 |
| 17. | I believe there is nothing more important than romantic relationships | 1 | 2 | 3 | 4 | 5 | 6 | 7 |
| 18. | I believe that making the wrong romantic decision would put me on a path of great misery. | 1 | 2 | 3 | 4 | 5 | 6 | 7 |
| 19. | It is almost impossible for me to leave a romantic relationship | 1 | 2 | 3 | 4 | 5 | 6 | 7 |
| 20. | For me, living without a romantic relationship is not living at all | 1 | 2 | 3 | 4 | 5 | 6 | 7 |

Relationship Assessment Scale)

|  | LOW |  |  |  | HIGH |
| --- | --- | --- | --- | --- | --- |
| 1. How well does your partner meet your needs? | 1 | 2 | 3 | 4 | 5 |
| 1. In general, how satisfied are you with your relationship? | 1 | 2 | 3 | 4 | 5 |
| 1. How good is your relationship compared to most? | 1 | 2 | 3 | 4 | 5 |
| 1. How often do you wish you hadn’t gotten into this relationship? | 1 | 2 | 3 | 4 | 5 |
| 1. To what extent has your relationship met your original expectations? | 1 | 2 | 3 | 4 | 5 |
| 1. How much do you love your partner? | 1 | 2 | 3 | 4 | 5 |
| 1. How many problems are there in your relationship? | 1 | 2 | 3 | 4 | 5 |

Experience in Close Relationship Scale

- Short Form (ECR-S)

# Instructions:

The following statements concern how you feel in romantic relationships. Please respond to each statement by indicating how much you agree or disagree.

|  | | Strongly Disagree | Disagree | Slightly Disagree | Neutral | Slightly Agree | Agree | Strongly Agree |
| --- | --- | --- | --- | --- | --- | --- | --- | --- |
| 1  2  3  4  5  6  7  8  9  10  11  12 | I worry about being abandoned. | **1** | **2** | **3** | **4** | **5** | **6** | **7** |
|  | I worry that other people won't care about me as much as I care about them. | **1** | **2** | **3** | **4** | **5** | **6** | **7** |
|  | I worry a fair amount about losing people close to me | **1** | **2** | **3** | **4** | **5** | **6** | **7** |
|  | I don't feel comfortable opening up to other people | **1** | **2** | **3** | **4** | **5** | **6** | **7** |
|  | I worry about being alone. | **1** | **2** | **3** | **4** | **5** | **6** | **7** |
|  | I feel comfortable sharing my private thoughts and feelings with other people | **1** | **2** | **3** | **4** | **5** | **6** | **7** |
|  | I need a lot of reassurance that I am loved by people close to me | **1** | **2** | **3** | **4** | **5** | **6** | **7** |
|  | If I can't get other people to show interest in me, I get upset or angry | **1** | **2** | **3** | **4** | **5** | **6** | **7** |
|  | I tell people close to me just about everything. | **1** | **2** | **3** | **4** | **5** | **6** | **7** |
|  | I usually discuss my problems and concerns with people close to me | **1** | **2** | **3** | **4** | **5** | **6** | **7** |
|  | I feel comfortable depending on other people | **1** | **2** | **3** | **4** | **5** | **6** | **7** |
|  | I don't mind asking other people for comfort, advice, or help. | **1** | **2** | **3** | **4** | **5** | **6** | **7** |

Obsessional Beliefs Questionnaire (OBQ-20)

This inventory lists different attitudes or beliefs that people sometimes hold. Read each statement carefully and decide how much you agree or disagree with it.

For each of the statements, choose the number matching the answer that *best describes how you think.* Because people are different, there are no right or wrong answers.

To decide whether a given statement is typical of your way of looking at things, simply keep in mind what you are like *most of the time.*

Use the following scale:

| 1 | 2 | 3 | 4 | 5 | 6 | 7 |
| --- | --- | --- | --- | --- | --- | --- |
| disagree | disagree | disagree | neither agree | agree | agree | agree |
| very much | moderately | a little | nor disagree | a little | moderately | very much |

In making your ratings, try to avoid using the middle point of the scale (4), but rather indicate whether you usually disagree or agree with the statements about your own beliefs and attitudes.

| 1. If I am not absolutely sure of something, I am bound to make a mistake | 1 | 2 | 3 | 4 | 5 | 6 | 7 |
| --- | --- | --- | --- | --- | --- | --- | --- |
| 2. In order to be a worthwhile person, I must be perfect at everything I do. | 1 | 2 | 3 | 4 | 5 | 6 | 7 |
| 3. Even if harm is very unlikely, I should try to prevent it at any cost. | 1 | 2 | 3 | 4 | 5 | 6 | 7 |
| 4. For me, having bad urges is as bad as actually carrying them out. | 1 | 2 | 3 | 4 | 5 | 6 | 7 |
| 5. If I don’t act when I foresee danger, then I am to blame for any consequences. | 1 | 2 | 3 | 4 | 5 | 6 | 7 |
| 6. In all kinds of daily situations, failing to prevent harm is just as bad as deliberately causing harm. | 1 | 2 | 3 | 4 | 5 | 6 | 7 |
| 7. For me, not preventing harm is as bad as causing harm. | 1 | 2 | 3 | 4 | 5 | 6 | 7 |
| 8. I should be upset if I make a mistake. | 1 | 2 | 3 | 4 | 5 | 6 | 7 |
| 9. For me, things are not right if they are not perfect. | 1 | 2 | 3 | 4 | 5 | 6 | 7 |
| 10. Having nasty thoughts means I am a terrible person. | 1 | 2 | 3 | 4 | 5 | 6 | 7 |
| 11. If I do not take extra precautions, I am more likely than others to have or cause a serious disaster. | 1 | 2 | 3 | 4 | 5 | 6 | 7 |
| 12. I am more likely than other people to accidentally cause harm to myself or to others. | 1 | 2 | 3 | 4 | 5 | 6 | 7 |

| 13. Having bad thoughts means I am weird or abnormal. | 1 | 2 | 3 | 4 | 5 | 6 | 7 |
| --- | --- | --- | --- | --- | --- | --- | --- |
| 14. Even when I am careful, I often think that bad things will happen. | 1 | 2 | 3 | 4 | 5 | 6 | 7 |
| 15. Having intrusive thoughts means I'm out of control. | 1 | 2 | 3 | 4 | 5 | 6 | 7 |
| 16. Avoiding serious problems (for example, illness or accidents) requires constant effort on my part. | 1 | 2 | 3 | 4 | 5 | 6 | 7 |
| 17. I must keep working at something until it's done exactly right. | 1 | 2 | 3 | 4 | 5 | 6 | 7 |
| 18. To me, failing to prevent a disaster is as bad as causing it. | 1 | 2 | 3 | 4 | 5 | 6 | 7 |
| 19. Having a bad thought is morally no different than doing a bad deed. | 1 | 2 | 3 | 4 | 5 | 6 | 7 |
| 20. No matter what I do, it won’t be good enough. | 1 | 2 | 3 | 4 | 5 | 6 | 7 |

| DAS S *Name: Date:* | | |
| --- | --- | --- |
| Please read each statement and circle a number 0, 1, 2 or 3 which indicates how much the statement applied to you *over the past week*.  There are no right or wrong answers.  Do not spend too much time on any statement. | | |
| *The rating scale is as follows:*  0  Did not apply to me at all  1  Applied to me to some degree, or some of the time  2  Applied to me to a considerable degree, or a good part of time  3  Applied to me very much, or most of the time | | |
| 1 | I found myself getting upset by quite trivial things | 0      1      2      3 |
| 2 | I was aware of dryness of my mouth | 0      1      2      3 |
| 3 | I couldn't seem to experience any positive feeling at all | 0      1      2      3 |
| 4 | I experienced breathing difficulty (eg, excessively rapid breathing, breathlessness in the absence of physical exertion) | 0      1      2      3 |
| 5 | I just couldn't seem to get going | 0      1      2      3 |
| 6 | I tended to over-react to situations | 0      1      2      3 |
| 7 | I had a feeling of shakiness (eg, legs going to give way) | 0      1      2      3 |
| 8 | I found it difficult to relax | 0      1      2      3 |
| 9 | I found myself in situations that made me so anxious I was most relieved when they ended | 0      1      2      3 |
| 10 | I felt that I had nothing to look forward to | 0      1      2      3 |
| 11 | I found myself getting upset rather easily | 0      1      2      3 |
| 12 | I felt that I was using a lot of nervous energy | 0      1      2      3 |
| 13 | I felt sad and depressed | 0      1      2      3 |
| 14 | I found myself getting impatient when I was delayed in any way (eg, lifts, traffic lights, being kept waiting) | 0      1      2      3 |
| 15 | I had a feeling of faintness | 0      1      2      3 |
| 16 | I felt that I had lost interest in just about everything | 0      1      2      3 |
| 17 | I felt I wasn't worth much as a person | 0      1      2      3 |
| 18 | I felt that I was rather touchy | 0      1      2      3 |
| 19 | I perspired noticeably (eg, hands sweaty) in the absence of high temperatures or physical exertion | 0      1      2      3 |
| 20 | I felt scared without any good reason | 0      1      2      3 |
| 21 | I felt that life wasn't worthwhile | 0      1      2      3 |

ROCI-Scenarios

***Instructions***

Below you will find everyday situations.  When reading each one of these, place yourself in the situation as if it was actually happening to you. Some of the situations may seem quite similar, so please pay attention to the details within each of the descriptions.

**After each situation you will find a series of questions.  Answer them as if you were really confronted with the situation.**

**(A) You are about to meet with your partner for lunch, suddenly the thought that you don't really love your partner pops up.**

| 1. To what extent do you feel an **urge to do something** about your concerns in this situation? |
| --- |
| 1 2 3 4 5 6 7 8 9 |
| \| Not at all \|  \|  \|  \| Moderately \|  \|  \|  \| Extremely \| \| --- \| --- \| --- \| --- \| --- \| --- \| --- \| --- \| --- \| \|  \|  \|  \|  \|  \|  \|  \|  \|  \| |

**(B) Your partner and you are with friends. Then, you feel the need to check whether the relationship between your partner and you feels "right".**

| 1. To what extent do you feel **discomfort** (e.g., anxious guilty, stressed, sad, uncomfortable, etc., ) in this situation? |
| --- |
| 1 2 3 4 5 6 7 8 9 |
| \| Not at all \|  \|  \|  \| Moderately \|  \|  \|  \| Extremely \| \| --- \| --- \| --- \| --- \| --- \| --- \| --- \| --- \| --- \| \|  \|  \|  \|  \|  \|  \|  \|  \|  \| |

**(C)   You are at home with your partner and feel the need to check whether your partner really loves you.**

| 1. To what extent do you feel **discomfort** (e.g., anxious guilty, stressed, sad, uncomfortable, etc., ) in this situation? |
| --- |
| 1 2 3 4 5 6 7 8 9 |
| \| Not at all \|  \|  \|  \| Moderately \|  \|  \|  \| Extremely \| \| --- \| --- \| --- \| --- \| --- \| --- \| --- \| --- \| --- \| \|  \|  \|  \|  \|  \|  \|  \|  \|  \| |

**(D) You are at school and your partner is at home. The doubt that your partner may not really love you suddenly comes up.**

| 1. To what extent do you feel an **urge to do something** about your concerns in this situation? |
| --- |
| 1 2 3 4 5 6 7 8 9 |
| \| Not at all \|  \|  \|  \| Moderately \|  \|  \|  \| Extremely \| \| --- \| --- \| --- \| --- \| --- \| --- \| --- \| --- \| --- \| \|  \|  \|  \|  \|  \|  \|  \|  \|  \| |

**(E) Your partner and you are on a date and you doubt your love for him/her.**

| 1. To what extent do you feel the **need to check whether you are suitable for each other**? |
| --- |
| 1 2 3 4 5 6 7 8 9 |
| \| Not at all \|  \|  \|  \| Moderately \|  \|  \|  \| Extremely \| \| --- \| --- \| --- \| --- \| --- \| --- \| --- \| --- \| --- \| \|  \|  \|  \|  \|  \|  \|  \|  \|  \| |

**(F) You and your partner are watching television. Then, you suddenly feel that you must remind yourself why you love my partner.**

| 1. To what extent do you feel **discomfort** (e.g., anxious guilty, stressed, sad, uncomfortable, etc., ) in this situation?s |
| --- |
| 1 2 3 4 5 6 7 8 9 |
| \| Not at all \|  \|  \|  \| Moderately \|  \|  \|  \| Extremely \| \| --- \| --- \| --- \| --- \| --- \| --- \| --- \| --- \| --- \| \|  \|  \|  \|  \|  \|  \|  \|  \|  \| |

**(G) At school, the thought that something is not quite right in your relationship with your partner pops up.**

| 1. To what extent do you feel an **urge to do something** about your concerns in this situation? |
| --- |
| 1 2 3 4 5 6 7 8 9 |
| \| Not at all \|  \|  \|  \| Moderately \|  \|  \|  \| Extremely \| \| --- \| --- \| --- \| --- \| --- \| --- \| --- \| --- \| --- \| \|  \|  \|  \|  \|  \|  \|  \|  \|  \| |

**(H) You are waiting for a phone call from your partner, suddenly the thought that your partner does not want to be with you pops into you mind.**

| 1. To what extent do you feel the **need to check whether you are suitable for each other**? |
| --- |
| 1 2 3 4 5 6 7 8 9 |
| \| Not at all \|  \|  \|  \| Moderately \|  \|  \|  \| Extremely \| \| --- \| --- \| --- \| --- \| --- \| --- \| --- \| --- \| --- \| \|  \|  \|  \|  \|  \|  \|  \|  \|  \| |

**(H) After a phone conversation with your partner, you begin to doubt your relationship.**

| 9. To what extent do you feel the **need to check whether you are suitable for each other**? |
| --- |
| 1 2 3 4 5 6 7 8 9 |
| \| Not at all \|  \|  \|  \| Moderately \|  \|  \|  \| Extremely \| \| --- \| --- \| --- \| --- \| --- \| --- \| --- \| --- \| --- \| \|  \|  \|  \|  \|  \|  \|  \|  \|  \| |


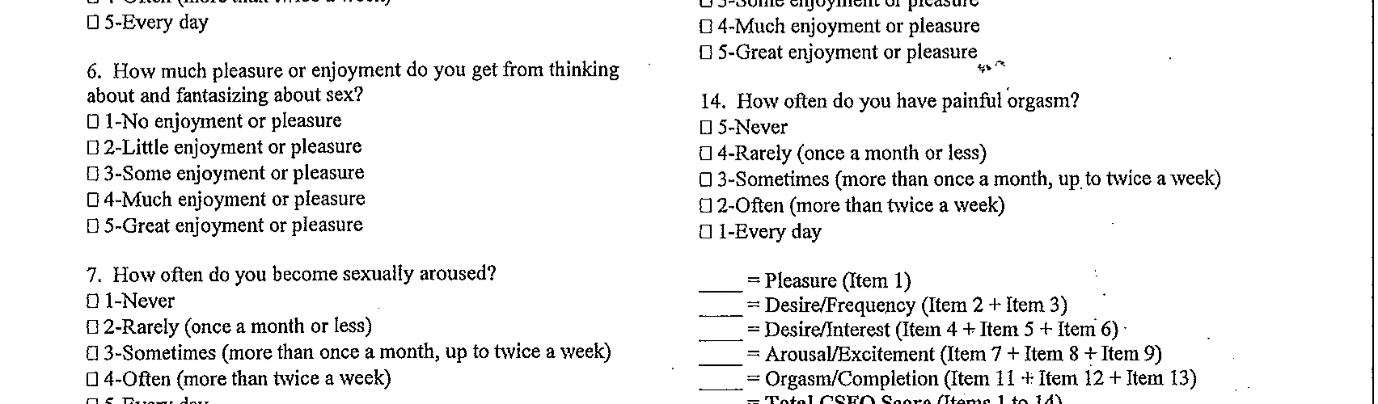

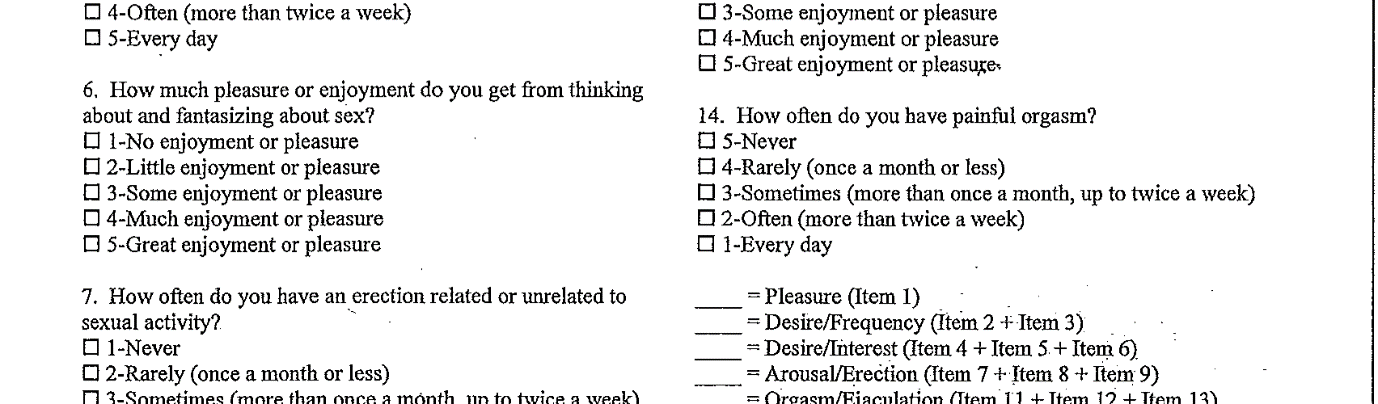

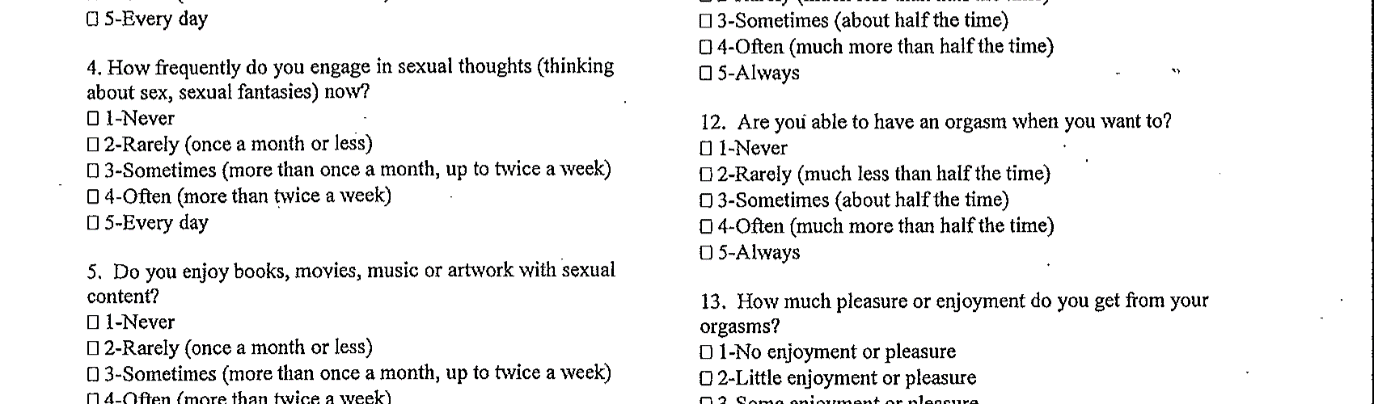

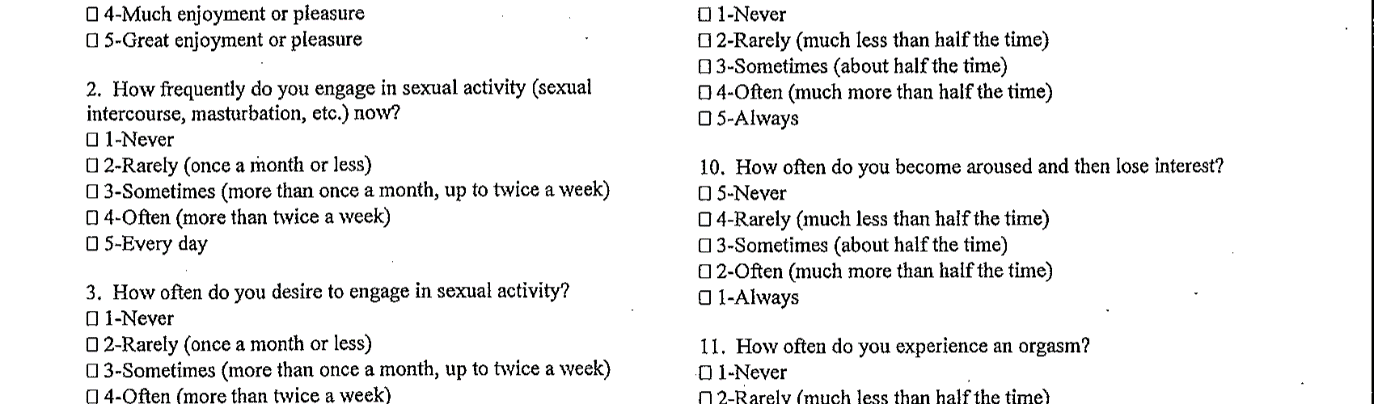

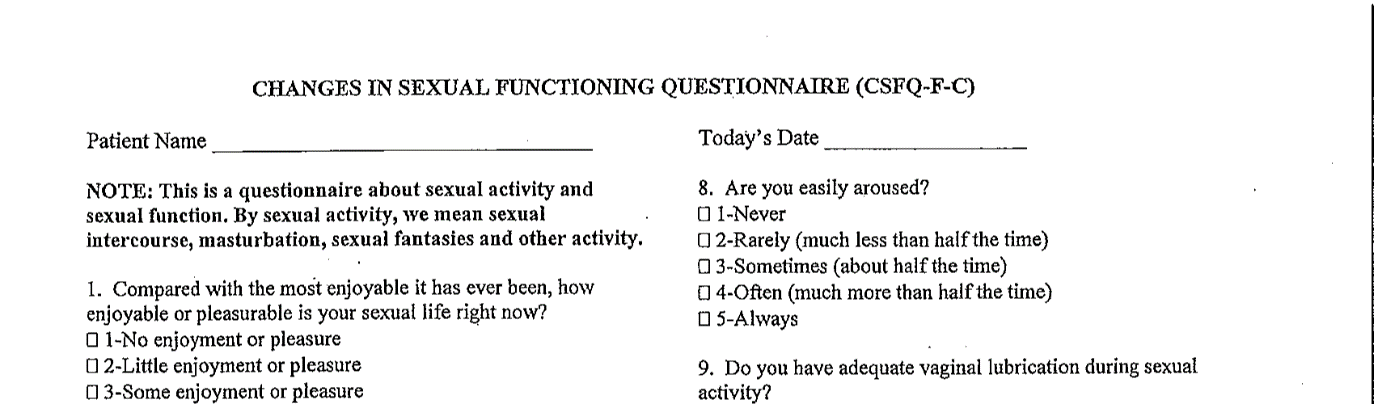

Supplement: Multimedia component 1 [file mmc1.docx]
