# Supplementary material for: Promoting couples’ resilience to relationship obsessive compulsive disorder (ROCD) symptoms using a CBT-based mobile application: A randomized controlled trial
Source: Heliyon. 2023 Oct 28;9(11):e21673. doi: 10.1016/j.heliyon.2023.e21673 (PMC10656241; doi:10.1016/j.heliyon.2023.e21673)
Supplement: Multimedia component 2 [file mmc2.docx]

**Online Supplementary Materials (OSM)**

| **Table 1.** Univariate effects of ROCI scores | | | | |
| --- | --- | --- | --- | --- |
|  | *df* | *F* | *p* | *η_p_^2^* |
| Time | 2, 202 | 1.41 | .25 | .01 |
| Group | 1, 101 | 3.91 | .051 | .04 |
| Gender | 1, 101 | 8.42 | .005 | .08 |
| Time × Group | 2, 202 | 5.17 | .006 | .05 |
| Time × Gender | 1.84, 185.98^1^ | 1.93 | .15 | .02 |
| Group × Gender | 1, 101 | .58 | .45 | .006 |
| Time × Group × Gender | 1.84, 185.98^1^ | .30 | .73 | .003 |
| Note: *ROCI: Relationship Obsessive–Compulsive Inventory.*  *All statistical tests were corrected for Type I error using the False Discovery Rate (Benjamini & Hochberg, 1995). In this case, significance was determined by p values less than .044 using this methodology.*  *^1^* *Greenhouse-Geisser correction* | | | | |

| **Table 2**. Univariate effects of PROCSI scores | | | | |
| --- | --- | --- | --- | --- |
|  | *df* | *F* | *p* | *η_p_^2^* |
| Time | 1.55, 156.57^1^ | 5.56 | .009 | .05 |
| Group | 1, 101 | 7.28 | .008 | .07 |
| Gender | 1, 101 | 2.63 | .11 | .03 |
| Time × Group | 1.55, 156.57^1^ | 8.91 | .001 | .08 |
| Time × Gender | 1.86, 187.35^1^ | .61 | .53 | .006 |
| Group × Gender | 1, 101 | .49 | .49 | .005 |
| Time × Group × Gender | 1.86, 187.35^1^ | 1.59 | .21 | .02 |
| Note: *PROCSI: Partner-Related Obsessive–Compulsive Symptoms Inventory.*  *All statistical tests were corrected for Type I error using the False Discovery Rate (Benjamini & Hochberg, 1995). In this case, significance was determined by p values less than .044 using this methodology.*  *^1^* *Greenhouse-Geisser correction* | | | | |

| **Table 3**. Univariate effects of ReCats scores | | | | |
| --- | --- | --- | --- | --- |
|  | *df* | *F* | *p* | *η_p_^2^* |
| Time | 2, 202 | 22.91 | .000 | .19 |
| Group | 1, 101 | 12.02 | .001 | .11 |
| Gender | 1, 101 | 3.51 | .06 | .03 |
| Time × Group | 2, 202 | 4.84 | .009 | .05 |
| Time × Gender | 1.88, 189.67^1^ | 1.99 | .14 | .02 |
| Group × Gender | 1, 101 | 4.82 | .03 | .05 |
| Time × Group × Gender | 1.88, 189.67^1^ | 1.49 | .23 | .02 |
| Note: *RECATS: Relationship Catastrophization Scale.*  *All statistical tests were corrected for Type I error using the False Discovery Rate (Benjamini & Hochberg, 1995). In this case, significance was determined by p values less than .044 using this methodology.*  *^1^* *Greenhouse-Geisser correction* | | | | |

| **Table 4**. Univariate effects of OBQ-20 scores | | | | |
| --- | --- | --- | --- | --- |
|  | *df* | *F* | *p* | *η_p_^2^* |
| Time | 2, 202 | 10.96 | .000 | .10 |
| Group | 1, 101 | 11.91 | .001 | .11 |
| Gender | 1, 101 | 2.19 | .14 | .02 |
| Time × Group | 2, 202 | 7.96 | .000 | .07 |
| Time × Gender | 2, 202 | 3.41 | .04 | .03 |
| Group × Gender | 1, 101 | 5.30 | .02 | .05 |
| Time × Group × Gender | 2, 202 | .10 | .90 | .001 |
| Note: *OBQ-20: short form of Obsessional Beliefs Questionnaire*  *All statistical tests were corrected for Type I error using the False Discovery Rate (Benjamini & Hochberg, 1995). In this case, significance was determined by p values less than .044 using this methodology.* | | | | |

| **Table 5**. Univariate effects of DASS-7 scores | | | | |
| --- | --- | --- | --- | --- |
|  | *df* | *F* | *p* | *η_p_^2^* |
| Time | 1.53, 154.26^1^ | 14.01 | .000 | .12 |
| Group | 1, 101 | 4.06 | .047 | .04 |
| Gender | 1, 101 | 5.71 | .02 | .05 |
| Time × Group | 1.53, 154.26^1^ | 3.34 | .051 | .03 |
| Time × Gender | 1.89, 190.78^1^ | .62 | .53 | .006 |
| Group × Gender | 1, 101 | .22 | .64 | .002 |
| Time × Group × Gender | 1.89, 190.78^1^ | .03 | .96 | .000 |
| Note: *DASS-7: Depression anxiety stress scale*  *All statistical tests were corrected for Type I error using the False Discovery Rate (Benjamini & Hochberg, 1995). In this case, significance was determined by p values less than .044 using this methodology.*  *^1^* *Greenhouse-Geisser correction* | | | | |

| **Table 6**. Univariate effects of ECR-S anxiety and avoidance scores | | | | |
| --- | --- | --- | --- | --- |
|  | ECR-S Anxiety | | | |
|  | *df* | *F* | *p* | *η_p_^2^* |
| Time | 2, 202 | .69 | .50 | .007 |
| Group | 1, 101 | .07 | .79 | .001 |
| Gender | 1, 101 | 21.14 | .000 | .17 |
| Time × Group | 2, 202 | 3.82 | .02 | .04 |
| Time × Gender | 2, 202 | 6.48 | .002 | .06 |
| Group × Gender | 1, 101 | .24 | .63 | .002 |
| Time × Group × Gender | 2, 202 | .05 | .95 | .001 |
|  | ECR-S Avoidance | | | |
|  | *df* | *F* | *p* | *η_p_^2^* |
| Time | 1.88, 189.84^1^ | .25 | .77 | .002 |
| Group | 1, 101 | 5.64 | .02 | .05 |
| Gender | 1, 101 | .67 | .42 | .007 |
| Time × Group | 1.88, 189.84^1^ | 5.33 | .007 | .05 |
| Time × Gender | 2, 202 | 1.63 | .20 | .02 |
| Group × Gender | 1, 101 | .01 | .93 | .000 |
| Time × Group × Gender | 2, 202 | 6.53 | .002 | .06 |
| Note: *ECR-S: short form of Experience in close relationship scale*  *All statistical tests were corrected for Type I error using the False Discovery Rate (Benjamini & Hochberg, 1995). In this case, significance was determined by p values less than .044 using this methodology.*  *^1^* *Greenhouse-Geisser correction* | | | | |

| **Table 7**. Univariate effects of RAS scores | | | | |
| --- | --- | --- | --- | --- |
|  | *df* | *F* | *p* | *η_p_^2^* |
| Time | 1.68, 169.80^1^ | 5.17 | .01 | .05 |
| Group | 1, 101 | 4.55 | .04 | .04 |
| Gender | 1, 101 | .02 | .90 | .000 |
| Time × Group | 1.68, 169.80^1^ | 7.80 | .001 | .07 |
| Time × Gender | 2, 202 | .03 | .98 | .000 |
| Group × Gender | 1, 101 | .080 | .78 | .001 |
| Time × Group × Gender | 2, 202 | .35 | .71 | .003 |
| Note: *RAS: Relationship assessment scale*  *All statistical tests were corrected for Type I error using the False Discovery Rate (Benjamini & Hochberg, 1995). In this case, significance was determined by p values less than .044 using this methodology.*  *^1^* *Greenhouse-Geisser correction* | | | | |

| **Table 8**. Univariate effects of CSFQ-14 scores | | | | |
| --- | --- | --- | --- | --- |
|  | *df* | *F* | *p* | *η_p_^2^* |
| Time | 1.64, 165.85^1^ | 3.63 | .04 | .04 |
| Group | 1, 101 | 3.75 | .056 | .04 |
| Gender | 1, 101 | 59.63 | .000 | .37 |
| Time × Group | 1.64, 165.85^1^ | 1.58 | .21 | .02 |
| Time × Gender | 1.75, 176.54^1^ | 1.02 | .35 | .01 |
| Group × Gender | 1, 101 | .04 | .85 | .000 |
| Time × Group × Gender | 1.75, 176.54^1^ | .29 | .72 | .003 |
| Note: *CSFQ-14: short form of Changes In Sexual Function Questionnaire*.  *All statistical tests were corrected for Type I error using the False Discovery Rate (Benjamini & Hochberg, 1995). In this case, significance was determined by p values less than .044 using this methodology.*  *^1^* *Greenhouse-Geisser correction* | | | | |
